# Unravelling the Supramolecular Driving Forces in the Formation of CO_2_-Responsive Pseudopeptidic Low-Molecular-Weight Hydrogelators

**DOI:** 10.3390/gels8060390

**Published:** 2022-06-20

**Authors:** Ferran Esteve, Alexis Villanueva-Antolí, Belén Altava, Eduardo García-Verdugo, Santiago V. Luis

**Affiliations:** 1 Departamento de Química Inorgánica y Orgánica, Universitat Jaume I, Av. Sos Baynat s/n, 12071 Castelló de la Plana, Spain; estevef@uji.es (F.E.); cepeda@uji.es (E.G.-V.); 2 Institute of Advanced Materials (INAM), Universitat Jaume I, 12071 Castelló de la Plana, Spain; aantoli@uji.es

**Keywords:** pseudopeptides, hydrogels, LMWG, supramolecular chemistry, responsive materials, CO_2_ absorption

## Abstract

A new family of C_2_-symmetric pseudopeptides with a high functional density for supramolecular interactions has been synthetized through the attachment of four amino acid subunits to a diamino aliphatic spacer. The resulting open-chain compounds present remarkable properties as low-molecular-weight hydrogelators. The self-assembled 3D networks were characterized by SEM analyses, observing regular nanofibres with 80–100 nm diameters. Spectroscopic and molecular modelling experiments revealed the presence of strong synergic effects between the H-bonding and π–π interactions, with the best results obtained for the homoleptic tetra-pseudopeptide derived from l-Phe. In addition, these bioinspired hydrogels possessed pH- and CO_2_-responsive sol–gel transitions. The formation of ammonium carbamate derivatives in the presence of carbon dioxide led to a detrimental change in its adequate self-assembly. CO_2_ desorption temperatures of ca. 70 °C were assigned to the thermodynamically favoured recovery of the supramolecular gel.

## 1. Introduction

Soft materials based on low-molecular-weight gelators (LMWG) have received significant attention in the last two decades, as they present some interesting features such as well-defined molecular weights, easy structural tuneability, and their capacity to form relatively regular nanostructures [1,2,3,4]. The strength of their 3D networks, and thus, the critical gelation concentration (CGC) of the corresponding supramolecular gels, is extremely dependent on the intermolecular non-covalent interactions present [5,6,7].

Gels formed in aqueous systems, i.e., hydrogels, are of special interest due to the wide variety of applications possible, ranging from tissue engineering [8,9] and controlled drug delivery [10,11,12] to nanoscale electronics [13] and actuators [14,15]. Hydrogelators based on amino acids are very attractive because of the highly tuneable characteristics of the amino acid fragments, their natural origin, and, accordingly, their potential for biocompatibility and biodegradability [16,17,18,19,20,21].

In this regard, C_2_-symmetric pseudopeptidic compounds displaying apolar spacers have shown remarkable gelating behaviours in different solvents [22], with the presence of additional functional components, such as urea fragments, significantly increasing the stability of the resulting supramolecular gels [23,24]. The introduction of polarfunctionalities in such pseudopeptides with apolar spacers has shown to afford efficient hydrogelators with pH-responsive self-assembling properties [25]. These results highlight the potential of using pseudopeptides as gelators due to the high flexibility of design that such molecules offer. Related approaches have allowed, for instance, the development of bioinspired hydrogels as controlled drug release systems [26].

Upon exposure to external stimuli, the dynamic nature of the different intermolecular interactions involved in the formation of a polymeric supramolecular structure enables reversible sol–gel transitions [27,28,29,30]. Unsurprisingly, many efforts have been devoted to the design of hydrogels that exhibit promising switchable properties on the basis of their different applications [31,32,33]. In this regard, CO_2_ is an ideal candidate for acting as a stimulus because it is a non-toxic, cheap, and abundant species. Therefore, several CO_2_-switchable soft materials have been precisely designed, leading to interesting advantages in the fields of chemosensors, absorbents, and drug delivery [34,35,36,37,38,39]. Nevertheless, the vast majority of the amino-based reported systems describe either the use of polymeric materials or the design of CO_2_-promoted LMWGs that are only suitable for gelation in organic solvents, thus hampering their use in biological applications [40]. An additional hurdle of amine-containing CO_2_ absorbents is the large energy consumption associated with the high temperatures required for desorption, stressing the need for the development of more efficient systems [41].

Taking these inspiring precedents into account, we report herein the preparation of open-chain C_2_-symmetric pseudopeptides containing four amino acid units within their structure. These simple compounds can be used to generate stable hydrogels in which self-assembly is governed by π–π interactions and H bonding [42,43]. The resulting supramolecular materials display promising properties for reversible, CO_2_-controlled hydrogelation. They present a rapid and isothermal CO_2_ absorption at room temperature, with subsequent CO_2_ desorption from the resulting ammonium carbamate derivative upon heating at temperatures of <80 °C, which defines a change in the self-assembly process.

## 2. Results and Discussion

### 2.1. Synthesis of the Pseudopeptidic Compounds and Gelation Properties of LMWGs

**Scheme 1:**
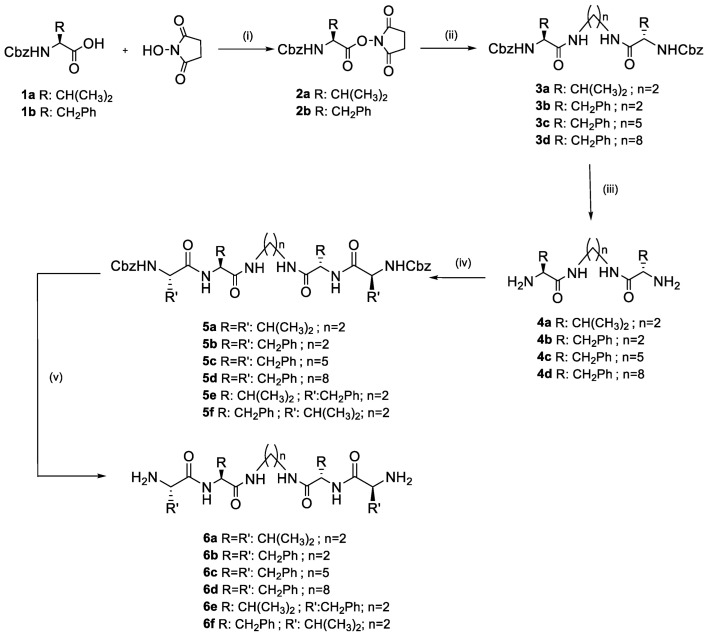
The synthesis of tetra-pseudopeptidic compounds. (i) DCC, 0 °C, 16 h; (ii) dry DME, ethylene diamine/1,5-diaminopentane/1,8-diaminooctane, r.t., 18 h; (iii) HBr/HAc (33%), 3 h/liquid-liquid extraction at pH = 10; (iv) dry DME, **2**, r.t., 18 h; and (v) H_2_, Pd/C, MeOH. All schemes follow the same formatting.

The synthesis of the different C_2_-symmetric tetra-pseudopeptides, **6a**–**f**, is shown in Scheme 1. Compounds **4a**–**d** were obtained following the previously reported synthetic procedure (Scheme 1) [44].

Briefly, the first step consisted of the synthesis of the activated esters of the l-amino acids, using DCC as the coupling agent. The resulting activated esters, **2a**,**b**, were reacted with the desired diamine to yield the N-Cbz-protected pseudopeptides, **3a**–**d**. The deprotection step to create the compounds **4a**–**d** relied on the use of a HBr/Hac (33%) solution. An additional coupling step with either **2a** or **2b** resulted in the N-protected tetra-pseudopeptides **5a**–**f**. The subsequent hydrogenation in the presence of palladium on activated carbon led to the desired deprotected tetra-pseudopeptides **6a**–**f**. The overall yields to produce **6a**–**f** from compounds **1a**,**b** ranged from 20% to 40%.

The gelation properties of the tetra-pseudopeptidic compounds **6a**–**f** were assayed in different aqueous mixtures and in water. As shown in Table 1, the nature of the amino acid side chain played a critical role in the self-assembly behaviour. As a matter of fact, the phenylalanine derivative **6b** formed gels in water mixtures and in water at 1 mg/mL (entries 2, 17, and 20, Table 1), while the valine-derived compound **6a** was not able to yield hydrogels under analogous conditions (entries 1, 18, and 19, Table 1). These results suggested that π–π interactions could be a major requirement for the 3D network to grow. Interestingly, when the gelation properties for compound **6b** were assayed in H_2_O:DMSO (90:10, *v*/*v*), an instantaneous gelation process was observed without the need for any external stimuli. On the other hand, when the experiments were performed in EtOH:H_2_O mixtures, sonication was always needed to obtain similar hydrogels.

The key role of aromatic interactions in the aggregation capacity of these pseudopeptides was confirmed by the results obtained for the heteroleptic compounds **6e** and **6f**, which contained only two aromatic groups in their side chains instead of the four displayed by **6b**. When 1 mg of **6e** or **6f** was dissolved in H_2_O:DMSO (90:10, *v*/*v*), a clear solution was obtained, thus revealing a lower gelating ability (entries 7 and 9, Table 1). When the concentration of the heteroleptic compounds was increased to 5 mg/mL, this triggered the formation of a weak hydrogel for **6f** (entry 10, Table 1**)**, whereas the **6e** sample still appeared as a clear solution (entry 8, Table 1). Interestingly, a weak gel was obtained when equimolar amounts of **6e** and **6f** (0.5 mg/mL each) were mixed, inferring that the two-component mixture was able to self-assemble in fibrillar aggregates (entry 11, Table 1). In view of these results, we also explored the hydrogelation properties of the precursors **4a**–**4e**, although their extremely low solubility in water hampered this approach.

**Figure 1:**
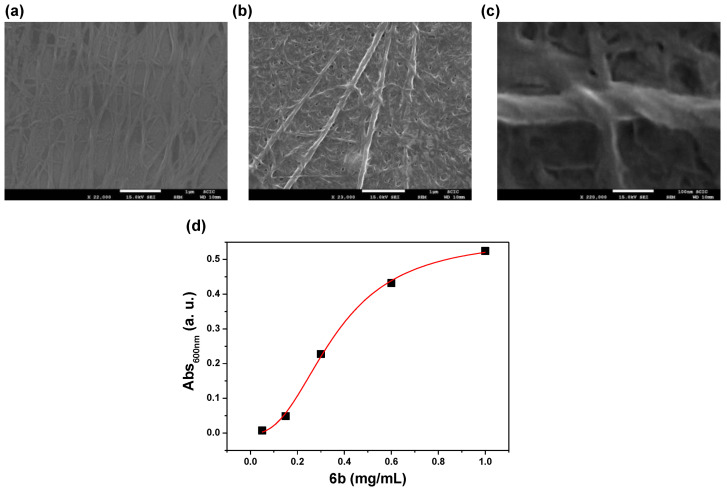
(**a**–**c**) SEM images for the dried sample of **6b** (1 mg/mL) in DMSO:H_2_O (10:90) and (**d**) UV absorption at 600 nm for **6b** at different concentrations in H_2_O:DMSO (90:10) [26]. The red line corresponds to the sigmoidal fitting calculated using Origin Software.

In addition, the effect of the aliphatic spacer length on the gelation ability was also evaluated. Previous studies on related pseudopeptides have demonstrated that, depending on the length of the alkyl aliphatic spacer, the amide groups can be in *-syn* or *-anti* disposition [45], which affects their self-assembly behaviour. Dissolving 1 mg of **6c**, which has three additional methylene groups in the spacer relative to **6b**, in 100 μL of DMSO and adding 900 μL of water led to the formation of a weak gel upon sonication. In order to obtain a stronger gel, 3 mg of **6c** was required (entries 3 and 4, Table 1). Compound **6d**, which contained an even longer spacer (*n* = 8), was only able to promote the formation of weak gels, even at 5 mg/mL (entries 5 and 6, Table 1). Thus, the compound with the shortest aliphatic spacer (*n* = 2), **6b**, presented the best gelation properties in aqueous media, most likely as a direct result of the optimal preorganization of the aromatic moieties of the side chains. SEM experiments provided additional insights into the microstructure of the hydrogels. Thus, SEM images for the dried gel of **6b** that was obtained in H_2_O:DMSO (90:10 *v*/*v*) allowed the presence of a well-defined interpenetrated network of regular nanofibers, displaying diameters of 80–100 nm, to be observed (Figure 1a–c) [46].

Vial inversion analyses allowed for the quantification of the critical gelation concentration (CGC), which is defined as the minimum amount of gelator required to gelate 1 mL of solvent at a given temperature [12]. The data presented in Table S1 permitted the assignment of a CGC lower than 0.6 mg/mL for compound **6b** (see Figure S1). This value demonstrated the remarkable gelation properties of **6b**, which belongs to the class of superhydrogelators [47]. UV–VIS spectroscopy was also used to determine the CGC for **6b** (Figure 1d). The formation of the gel could be straightforwardly monitored by measuring the absorption changes at 600 nm. A sigmoidal curve was observed with an inflexion point at ca. 0.4 mg/mL. This concentration was in good agreement with the value determined by the vial inversion method.

**Figure 2:**
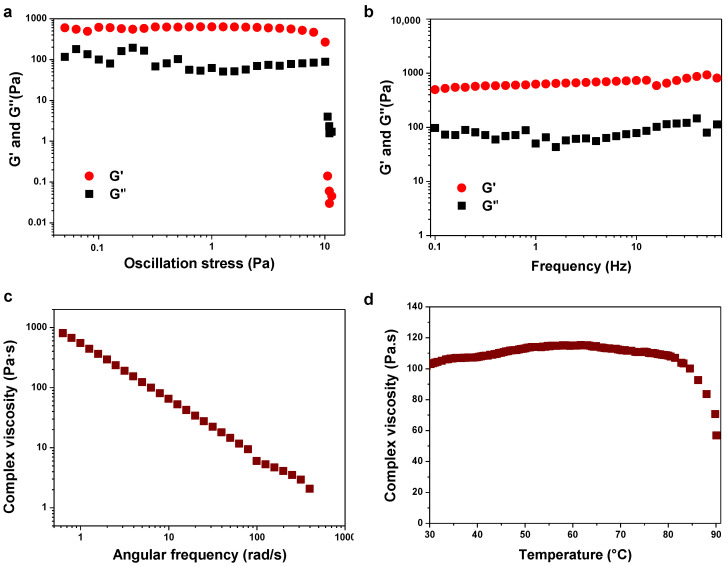
Rheological measurements for the hydrogel formed from **6b** (1 mg/mL, H_2_O:DMSO (90:10 *v*/*v*)). (**a**) Amplitude sweeps with 1 Hz at 25 °C; (**b**) frequency sweeps with 0.1 Pa strain at 25 °C; (**c**) complex viscosity vs. angular frequency; and (**d**) the dependence of the complex viscosity on the temperature using a heating rate of 0.2 °C/min and a constant frequency of 1 Hz and 0.1% strain.

The thermal stability of the gel formed from **6b** (H_2_O:DMSO (90:10 *v*/*v*), 1 mg/mL) was likewise investigated using the vial inversion test (Table S2). The hydrogel was stable until 85 °C, at which point the soft material started to lose its viscosity. This finding was attributed to some evaporation of the water present in the initial solvent mixture. The mechanical properties of this hydrogel (H_2_O:DMSO (90:10 *v*/*v*)) were studied using rheological measurements. A typical LMWG presents a storage and a loss modulus (G′ and G′′, respectively) which are frequency independent, and will break under relatively low strain [48]. Figure 2 displays the rheological results obtained for **6b** (1 mg/mL). The storage modulus (G′) of the gel was much higher than the loss modulus (G′′) over the whole range of the frequency sweep, which is characteristic of gel-like materials (Figure 2a). The strain sweep measurement (Figure 2b) showed that both the storage and loss moduli of the gel remained constant up to 10 Pa of stress (3.6% strain), corresponding to the so-called linear viscoelastic region (LVER). Furthermore, the average complex viscosity of the hydrogel was quantified, obtaining a value of 113 Pa·s. The complex viscosity dependence with the angular frequency was also evaluated, observing a linear relationship with the expected negative slope (Figure 2c). By heating the sample prepared in situ on the Peltier geometry, it was possible to evaluate the effect of temperature on the mechanical properties of the pseudopeptidic soft material. For instance, by observing the complex viscosity trend with temperature, a linear range was obtained from 25 to 85 °C (Figure 2d). When the temperature reached values higher than 83 °C, a clear decrease in the viscosity was observed, indicating that the sample was losing its gel-like properties. Hence, the critical gelation temperature (CGT) was identified as 83 °C, which is a value within the same range obtained by the vial inversion method (see Table S2). This material can be classified as a thixotropic gel because, upon shaking the vial, the sample became much more fluid than in its static state; furthermore, after resting the vial for 24 h, the gel could be restored. The same rheological experiments were also performed for **6b** at higher gelator concentrations (5 mg/mL in H_2_O:DMSO (90:10 *v*/*v*)). Not surprisingly, the resulting gel demonstrated a higher complex viscosity, with an average value of 1640 Pa·s, and higher G′ and G′′ values. This stronger gel was able to resist oscillation strains of ca. 40 Pa, corroborating the tougher properties of the gel at higher concentrations (Figure S2).

Next, we decided to analyse whether an external stimulus could induce a gel–sol transition [49]. Considering the functional groups present in the chemical structure of **6b**, the pH appeared to be a good candidate, as the pseudopeptide presented terminal amino groups (basic) and amide groups (slightly acidic). Therefore, vial inversion tests were carried out on the samples at different pH values, which were controlled by commercially available buffers (Figure S3). The results, presented in Table 2, revealed that the higher the pH was, the more favourable was the formation of the gel. It must be mentioned that the chemical structure of the LMWG was very stable and was not expected to degrade under the assayed conditions. Notwithstanding, these kinds of compounds have been observed to degrade by proteases under biological conditions, which represents an interesting feature in terms of their biodegradability [26].

These pH-dependent results indicated the participation of the terminal amino groups in the formation of the non-covalent network, since when these groups were in their fully protonated form, a completely transparent solution was obtained. Interestingly enough, some crystallization could be observed in the sample at a pH of 1 after aging the vial for one week. The SEM images of the resulting crystalline solid revealed the formation of fibre-like crystals, which were likely the result of the formation of diprotonated ammonium salts for **6b** (Figure S4). Therefore, the protonation of the primary amines led to a soluble ammonium salt that hampered the formation of the 3D supramolecular network. It must be noted, however, that **6b** was able to form weak gels even at a physiological pH (Table 2, vial 4), which opens a wide scope for future applications in areas such as drug delivery.

### 2.2. Supramolecular Driving Forces

**Figure 3:**
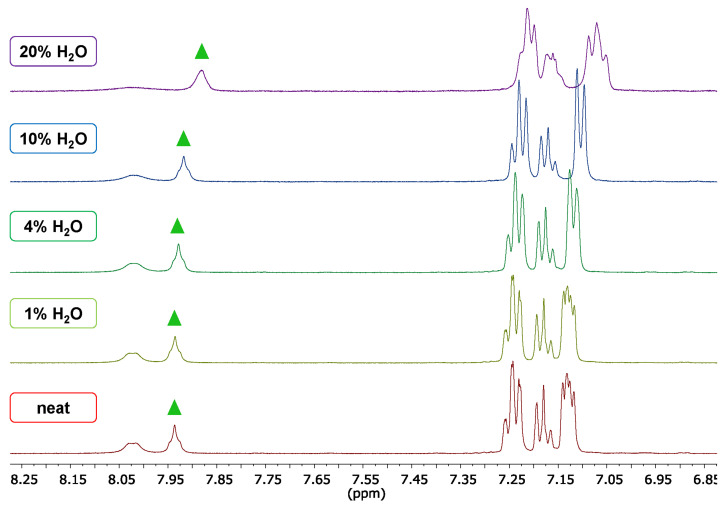
Partial ^1^H NMR (500 MHz, DMSO-*d_6_*) spectra for the titration of **6b** (3 mM) with increasing amounts of water. The water content is indicated as *v*/*v* (%). The signal for the central amide groups has been marked with green triangles.

To obtain relevant information about the supramolecular interactions that governed the formation of these soft materials, a series of ^1^H NMR and theoretical studies were undertaken. First of all, increasing amounts of water were added to a 3 mM solution of **6b** in DMSO-d_6_, and ^1^H NMR spectroscopy was used to observe some relevant changes in the characteristic signals of the pseudopeptide. Indeed, most of the peaks of **6b** broadened with the addition of water, suggesting the formation of oligomeric aggregates. An upfield shift was observed for the proton signals of the central amide groups (green triangles in Figure 3), indicating a different involvement of hydrogen bonding in the supramolecular aggregates.

In a similar manner, the protons corresponding to the aromatic moieties experienced a shift towards a lower δ. This was especially noticeable for the protons assigned to the aromatic ring of the central amino acid scaffolds, suggesting their participation in hydrophobic interactions. In addition, the methylene protons of the aliphatic central spacer and the benzylic protons also experienced a different electronic environment in the aggregates, as evidenced by the upfield shift for the central methylene protons (orange discontinuous lines, Figure S5).

In terms of the signals for the protons of the water molecules, a progressive downfield evolution could be observed, shifting from 3.35 to 3.93 ppm (Δδ = + 0.58 ppm) when the amount of water reached 20% *v*/*v* (purple discontinuous lines, Figure S5). This change in the electronic environment was likely the result of water entrapment in the three-dimensional network that was formed, thus increasing the H bonding. Interestingly, analogous experiments performed for **6f** revealed less-pronounced shifts for both the water molecules and the signals from the heteroleptic pseudopeptide (Figures S6 and S7). As a matter of fact, the signal for water shifted by Δδ = +0.28 ppm. Although the aromatic protons of **6f** also shifted towards lower δ values in the presence of water, this shift was much less significant than the one observed for **6b** (Figure S7). This indicates that the homoleptic tetra-phenylalanine-derived pseudopeptide promoted higher efficiencies in forming the supramolecular aggregates, in good accordance with the trends found in gelation studies (see, for instance, entries 2 and 9, Table 1).

**Figure 4:**
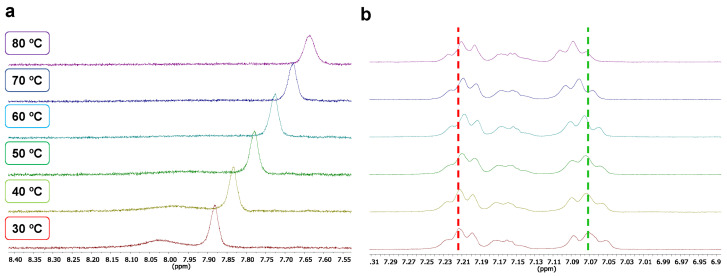
Partial ^1^H NMR (500 MHz, DMSO-*d_6_*) spectra for the variable-temperature experiments of **6b** (3 mM, H_2_O:DMSO, 20:80); (**a**) amide group region and (**b**) aromatic region.

Variable-temperature ^1^H NMR experiments corroborated the fundamental role of intermolecular hydrogen-bonding and π–π interactions in the self-assembly of the pseudopeptides. Important conformational information was gathered when a sample containing a 3 mM solution of **6b** in H_2_O:DMSO (20:80) was heated from 30 to 80 °C. As expected, the proton signals of the amide groups and water experienced an upfield shift as the temperature increased (*ca*. Δδ = −0.24 and −0.35 ppm for NH_amide_ and H_2_O, respectively), suggesting their participation through hydrogen-bonding interactions within the supramolecular network of aggregates (Figure 4a and Figure S8).

A different scenario was observed on behalf of the signals for the aromatic units. The multiplet appearing at 7.21 ppm underwent a minor upfield shift, as marked with a discontinuous red line in Figure 4b. On the other hand, a more pronounced change was noted for the signal appearing at 7.07 ppm at 30 °C, but with the opposite direction than the one found at 7.21 ppm. This trend is in good agreement with interconnected π systems, where one of the aromatic units acts as the π-donor and the other one as the π-acceptor [50]. Additionally, the protons of the chiral carbon centres, the methylene protons of the central spacer, and the benzylic protons experienced a small shift (Δδ = +0.03 ppm, discontinuous orange lines, Figure S8).

**Figure 5:**
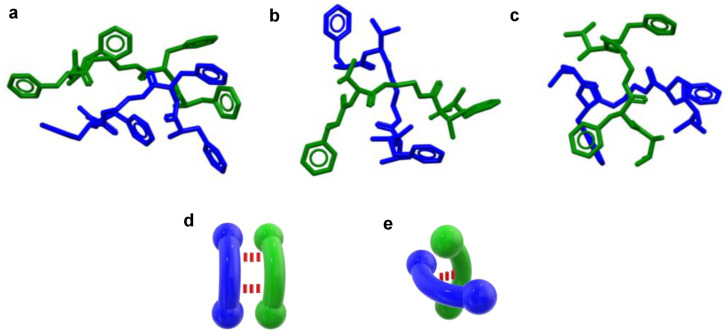
The most stable conformations (Spartan08′, MMFFaq) obtained for the dimers of (**a**) **6b**, (**b**) **6e**, and (**c**) **6f**; (**d**) a representation of the parallel β-sheet; and (**e**) the tangled dimeric assemblies.

Molecular modelling for **6b**, **6e**, and **6f** unravelled the importance of synergic π–π interactions, which were only observed in the case of **6b**. All the models were based on the molecular mechanics at the MMFFaq level of theory, taking into account the water solvation (see experimental section for more details). The most stable conformation for the dimeric species was calculated as a simple model to surmise whether each pseudopeptide could form larger aggregates by means of non-covalent forces. The modelized structures for the tetra-pseudopeptidic dimers revealed noteworthy disparities. For instance, the two modelized molecules of **6b** adopted a β-sheet spatial disposition that was stabilized by means of intermolecular hydrogen bonds involving the central amide groups of the pseudopeptidic scaffolds. Additionally, the hydrophobic aromatic units were located in the outer part of the molecules, intermolecularly interacting through π–π forces (Figure 5a). Therefore, the adopted conformation allowed for the construction of bigger self-assembled supramolecular structures, leading to the formation of fibre-like aggregates (Figure 5d). On the other hand, the modelized dimers for the heteroleptic tetra-pseudopeptides rather embraced a tangled conformation, only stabilized through intermolecular hydrogen bonding between the amide groups, which precluded an efficient extension of the network to form gels (Figure 5b,c,e). 

The non-covalent interactions governing the gel formation for **6b** were studied in greater detail. The four amide groups (presenting with an -*anti* disposition) of each of the pseudopeptidic molecules were highly interconnected with the analogues of the neighbouring molecule, leading to the formation of parallel hydrogen bonds (Figure S9a). The average distance between the O···N atoms involved in the interaction was 2.780 Å (90% vdW_N,O_) [51], suggesting a strong interaction even in a competitive polar medium. In terms of the hydrophobic interactions between the dimeric species, four different intermolecular edge-to-face π–π interactions were detected (Figure S9b). In addition, weak intramolecular NH···π forces were identified between the phenyl rings of the phenylalanine side chains and the acidic NH proton of the amide groups, further stabilising the whole system (Figure S9c) [52].

### 2.3. CO_2_ Stimulus Responsiveness

In view of these promising results, we envisaged that the soft materials could experience a reversible gel-to-sol transition triggered by carbon dioxide. It is well-known that the solubility of carbon dioxide in water leads to a noteworthy decrease in the pH, reaching values between 3.5 and 4 [53]. In addition, amines have been widely used as chemical absorbents of carbon dioxide [54]. The interaction of CO_2_ with unhindered primary amines is known to generate ammonium carbamate species, because primary amines are more basic and nucleophilic than water [55,56].

We performed ^1^H NMR analyses using DMSO-*d_6_* as the solvent to obtain information about the formation of ammonium carbamate products [57]. For comparison, the spectrum of the diprotonated (**6b**·2HCl) species in DMSO was also recorded. Significant differences were observed when comparing the spectra of freshly prepared **6b** with the one of (**6b**·2HCl) and that of **6b** after bubbling a CO_2_ balloon for 5 min (Figure S10). For **6b**–CO_2_, the downfield shift for the proton of the stereogenic carbon (from δ = 3.42 to 4.10 ppm), together with the appearance of new signals at 6.62, 8.24, and 8.59 ppm, could indicate the formation of the ammonium carbamate derivative. While the methylene protons of the central spacer shifted towards lower δ values for (**6b**·2HCl), no shift was observed for **6b**–CO_2_. Furthermore, a significant downfield shift of the aromatic protons and an increase in the anisochrony of the benzylic protons was observed for **6b**–CO_2_, with the latter being attributed to the lack of free rotation. As expected, the signals of these CO_2_-derived species decreased with time, even at room temperature, indicating that the interaction with carbon dioxide was reversible (see Figure S11). This quite fast dynamic equilibrium in the NMR tube precluded its study by ^13^C NMR. Two main approaches have been established in the literature for the formation of carbamate derivatives from diamino compounds: intramolecular asymmetric carbamate salts (Figure S12a), and dimeric symmetric carbamate species (Figure S12b) [55]. As the ^1^H NMR spectrum of **6b** in the presence of CO_2_ did not reflect a complex mixture of asymmetric signals, the formation of the dimeric symmetric ammonium carbamate species could be considered. 

**Figure 6:**
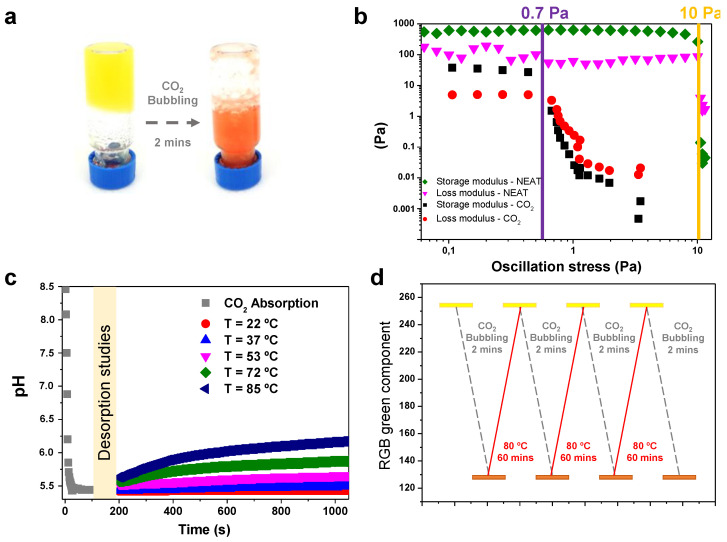
(**a**) A vial inversion test for the methyl red with the samples before (left) and after (right) CO_2_ incorporation (1 mg of **6b** in 1 mL of H_2_O(methyl red):DMSO (90:10)). (**b**) The storage and loss modulus representation for neat (green and pink, respectively) and CO_2_-rich (black and red, respectively) samples; the oscillation stress required for the gel-to-liquid transition is highlighted in purple for the CO_2_-rich sample and in orange for the freshly prepared **6b** hydrogel. (**c**) CO_2_ absorption/desorption results obtained via in-line monitorization of the pH for the **6b** gel (1 mg in 1 mL of H_2_O:DMSO, 90:10). (**d**) Cycles for the CO_2_ absorption/desorption–hydrogel depletion/formation obtained for **6b** by measuring the RGB green component observed (1 mg of **6b** in 1 mL of H_2_O:DMSO, 90:10).

When CO_2_ was bubbled into the **6b** gel sample (1 mg/mL in H_2_O:DMSO, 90:10 *v*/*v*), a transition from a gel to a weak gel was clearly observed by vial inversion, and the gel could not be restored after resting the vial for 24 h. The pH before and after the CO_2_ adsorption was 8.5 and 5.4, respectively. Thus, by selecting an appropriate pH indicator, it was possible to design a colorimetric probe for the gel–sol transition. The gelation of an aqueous solution of methyl red in DMSO (10% *v*/*v*) with **6b** (1 mg/mL) led to the expected yellowish gel (Figure 6a—left). After bubbling in the carbon dioxide, a change in the colour of the sample was observed, shifting from yellow to orange (Figure 6a—right). In addition, when comparing the rheological properties of a freshly prepared **6b** gel (1 mg/mL in H_2_O:DMSO 90:10 *v*/*v*) and the sample after CO_2_ absorption, the storage modulus (G′) decreased by more than one order of magnitude. The complex viscosity presented an average value of 5.2 Pa·s, which is more than 20 times smaller than the viscosity of the original gel (113 Pa·s). The formation of a weaker CO_2_-absorbed soft material was also corroborated with the values of the oscillation strain required for breaking down the gel. Whereas an oscillation strain of 10 Pa was necessary for the gel–liquid transition in the freshly prepared gel, a strain of only 0.7 Pa was necessary for the ammonium carbamate-derived sample (Figure 6b).

Generally, the desorption of CO_2_ is controlled by temperature for most of the systems reported to date. This is the consequence of ammonium carbamate decomposition at high temperatures, which releases carbon dioxide and the free amino groups [58]. We speculated whether our bioinspired materials could promote hydrogel recovery upon increasing temperatures. The CO_2_ absorption was conducted at 25 °C under atmospheric conditions and the desorption was carried out at temperatures between 30 and 80 °C. To precisely study the reversibility of the system, we determined the desorption temperature by in situ pH measurements for the sample. The pH of the gel drastically decreased from 8.5 to 5.4 in less than 100 s upon CO_2_ bubbling. At the same time, the viscosity of the system was clearly reduced after the addition of carbon dioxide. Once the gel had been depleted, we studied the pH variations at different temperatures, providing an insight into the CO_2_ desorption efficiency (Figure 6c). Interestingly, at temperatures > 70 °C, a significant increase in pH was observed, indicating the decomposition of the carbamate species. To our delight, this system was thus able to promote CO_2_ desorption at relatively low temperatures, considering that the ammonium carbamate species were formed from primary amino groups [41]. The efficient CO_2_ release was assigned to the thermodynamically favoured recovery of the supramolecular hydrogel.

Subsequently, we designed a suitable cycle for studying the hydrogel destruction/formation using CO_2_ as an external stimulus, obtaining promising results in terms of reversibility (Figure 6d). The experiment relied on simple colorimetric analyses using methyl orange as a pH indicator. An orange weak gel was observed after the CO_2_ absorption, resulting in a RGB green component value of 128. Contrarily, a yellowish colour (RGB green component = 255) was observed when the hydrogel was recovered after heating at 80 °C for 60 min.

Hence, the formation of the carbamate species clearly affected the outcome of the self-assembly of the pseudopeptide. We calculated the most stable conformation for the proposed ammonium carbamate symmetric dimeric derivatives at the MMFFaq level of theory. Interestingly, although some intermolecular hydrogen-bonding interactions were found between the amide groups and the carbamate scaffolds, only one intermolecular π–π interaction was detected, which supported the hypothesis that such aromatic forces are essential for achieving a supramolecular 3D network (Figure S13a). The dimeric ammonium carbamate derivative displayed a tight electrostatic interaction between the terminal ammonium and carbamate groups (Figure S13b), leading to the formation of discrete supramolecular architectures. Furthermore, the salts formed upon exposure to carbon dioxide possessed a higher solubility in water, decreasing the self-assembly efficiency (Figure S13c).

## 3. Conclusions

Novel open-chain pseudopeptides were successfully synthesized. These bioinspired species demonstrated promising properties as low-molecular-weight hydrogelators, with CGC values as low as 0.6 mg/mL. The nature of the supramolecular interactions governing gel formation was elucidated, revealing a strong cooperativity between hydrogen-bonding and π–π forces. Modifications to the side chain composition and the aliphatic spacer length allowed for the modulation of the properties of the final materials. The presence of terminal amino groups in their basic form was found to be crucial for efficient self-assembly. Sol–gel transitions could be easily triggered by pH modification, obtaining the toughest materials under basic conditions (pH > 8). In addition, the high tendency of primary amines to react with carbon dioxide permitted the CO_2_ responsiveness of these soft materials to be assayed. In this regard, remarkable results were obtained with desorption temperatures of ca. 70 °C, which were assigned to the thermodynamically favoured recovery of the supramolecular gel. 

## 4. Materials and Methods

### 4.1. General

The reagents and solvents were purchased from commercial suppliers and used without further purification. Deionized water was obtained from MilliQ (Burlington, MA, USA).

The NMR experiments were carried out on a Varian INOVA 500 spectrometer (Mundelein, IL, USA, 500 MHz for ^1^H and 125 MHz for ^13^C), on a Bruker 400 (400 MHz for ^1^H and 100 MHz for ^13^C), or on a Bruker 300 (300 MHz for ^1^H and 75 MHz for ^13^C). Chemical shifts were reported in parts per million using the solvent’s residual peak as the reference.

ATR FT-IR spectra were acquired using a JASCO 6200 device with a MIRacle Single Reflection ATR Diamond/ZnSe accessory. The samples were directly deposited onto the ATR sample holder, and the FT-IR spectra were collected. The raw IR data were processed with JASCO spectral manager software (Version 2).

UV–Vis absorption measurements were carried out on a Hewlett–Packard 8453 spectrophotometer (Palo Alto, CA, USA) with a controlled temperature system.

### 4.2. Gelation Experiments

In a typical experiment, a weighted amount of the LMWG was mixed with 1 mL of the selected solvent in a 1.5 mL glass vial. Gelation was defined when a homogeneous mixture was obtained that exhibited no gravitational flow upon inversion of the vial. For organic/water solvent mixtures, the LMWG was first dissolved in the corresponding amount of the organic solvent (DMSO or EtOH) and MilliQ water was subsequently added.

### 4.3. Rheological Experiments

The different gels were characterized using a controlled stress AR-2000 rheometer from TA Instruments (New Castle, DE, USA). A Peltier holder with a plate geometry (60 mm diameter, 500 μm gap) was used for all samples. Frequency sweeps were performed in the angular frequency range 0.1–100 rad s^−1^ with the instrument in oscillatory mode at 25 °C. Strain sweeps were performed using a frequency of 1 rad s^−1^ in an amplitude sweep range of 0.01–80% with the instrument in oscillatory mode at 25 °C.

### 4.4. Molecular Modelling

The lowest energy conformations for the different species considered were calculated at the MMFFaq level of theory using Spartan08 [59]. The stationary points were confirmed by subsequent frequency calculation. All vibrational frequencies were positive. See the supporting information for the Cartesian coordinates.

### 4.5. Synthetic Protocols

The C_2_-symmetrical bis(aminoamides) (4) were prepared as previously reported [44]. 

#### 4.5.1. Synthesis of Cbz-N Protected Tetra-Pseudopeptidic Intermediates (5)

##### Synthesis of **5a**

The compound **2a** (7.741 g, 10.686 mmol) was dissolved in anhydrous DME (150 mL) at 0 °C in a two-necked round-bottomed flask (500 mL) using a N_2_ atmosphere. A DME solution (150 mL) of **4a** (3.02 g, 11 mmol) was then added dropwise. The reaction mixture was stirred at 0 °C for 1 h and at 25 °C for 18 h. Subsequently, the white suspension was heated for 6 h at 40–50 °C. The resultant white precipitate was filtered off and washed with cold water and cold methanol. The solid product was dried overnight under reduced pressure at 50 °C to yield **5a** as a white solid. The yield was 7.09 g (9.79 mmol, 89.0%). The product was used for the subsequent step without further purification.

##### Synthesis of **5b**

Intermediate **5b** was successfully synthesized following the same procedure as described for **5a**, but using **2b** instead of **2a** and **4b** instead of **4a**. The yield was 3.473 g (3.804 mmol, 87.0%). The product was used for the subsequent step without further purification.

##### Synthesis of **5c**

Intermediate **5c** was successfully synthesized following the same procedure as described for **5a**, but using **2b** instead of **2a** and **4c** instead of **4a**. The yield was 3.186 g (3.330 mmol, 76.9%). The product was used for the subsequent step without further purification.

##### Synthesis of **5d**

Intermediate **5d** was successfully synthesized following the same procedure as described for **5a**, but using **2b** instead of **2a** and **4d** instead of **4a**. The yield was 2.209 g (2.192 mmol, 81.6%). The product was used for the subsequent step without further purification.

##### Synthesis of **5e**

Intermediate **5e** was successfully synthesized following the same procedure as described for **5a**, but using **2b** instead of **2a**. The yield was 4.931 g (6.001 mmol, 90.9%). The product was used for the subsequent step without further purification.

##### Synthesis of **5f**

Intermediate **5f** was successfully synthesized following the same procedure as described for **5a**, but using **4b** instead of **4a**. The yield was 1.238 g (1.506 mmol, 63.2%). The product was used for the subsequent step without further purification.

#### 4.5.2. Synthesis of Tetra-Pseudopeptidic Compounds (6)

##### Synthesis of **6a**

The compound **5a** (0.676 g, 0.932 mmol) was suspended in methanol (150 mL) in a 250 mL two-necked round-bottomed flask using an ultrasonic bath. After obtaining a N_2_ atmosphere, Pd/C (0.198 g, 0.092 mmol) was poured into the flask while nitrogen was being flushed. The nitrogen balloons were then replaced by ones filled with hydrogen and the mixture was stirred for 24 h. The suspension was filtered through a Celite^®^ layer, and the adsorbent was washed with methanol. The final solution was evaporated under reduced pressure to yield pure **6a** as a white solid. The yield was 0.346 g (0.758 mmol, 81.3%); the mp was 228–229 °C; [α]_D_^25^ = −7.32° (*c* = 0.1, DMSO); ATR-FTIR: 3278, 3050, 1634, and 1537 cm^−1^; ^1^H NMR (400 MHz, CD_3_OD) *δ =* 0.85–0.99 (m, 24H), 1.95 (m, 4H), 3.08 (t, *J* = 14.94, 14.94 Hz, 2H), 3.17 (dd, *J* = 5.46, 14.16 Hz, 2H), 3.24 (d, *J* = 6.55 Hz, 2H), and 4.01 (d, *J* = 7.07 Hz, 2H); ^13^C{^1^H} NMR (75 MHz, CD_3_OD) *δ* = 16.1, 17.3, 18.4, 18.5, 30.4, 31.8, 38.6, 58.8, 60.1, 172.6, and 175.8; HRMS (ESI/Q-TOF) m/z: [M + H]^+^ calculated for C_22_H_44_N_6_O_4_ 457.3502; found 457.3498. 

##### Synthesis of **6b**

The compound **6b** was successfully synthesized following the same procedure as described for **6a**, but using **5b** instead of **5a**. The yield was 0.331 g (0.510 mmol, 82.5%); the mp was 193–195 °C; [α]_D_^25^ = −3.42° (*c* = 0.05, DMSO); ATR-FTIR: 3282, 3042, 1648, and 1535 cm^−1^; ^1^H NMR (300 MHz, CD_3_OD) *δ =* 2.41–3.13 (m, 12H), 3.40–3.51 (m, 2H), 4.36–4.48 (m, 2H), and 6.95–7.29 (m, 20H); ^13^C{^1^H} NMR (75 MHz, CD_3_OD) *δ* = 37.6, 38.7, 40.3, 54.5, 56.0, 126.4, 126.5, 128.1, 128.2, 129.0, 129.0, 136.9, 137.3, 172.3, and 175.0; HRMS (ESI/Q-TOF) m/z: [M + H]^+^ calculated for C_38_H_44_N_6_O_4_ 649.3502; found 649.3508.

##### Synthesis of **6c**

The compound **6c** was successfully synthesized following the same procedure as described for **6a**, but using **5c** instead of **5a**. The yield was 1.269 g (1.837 mmol, 78.4%); the mp was 152–154 °C; [α]_D_^25^ = −6.21° (*c* = 0.05, DMSO); ATR-FTIR: 3283, 3042, 1639, and 1529 cm^−1^; ^1^H NMR (400 MHz, CD_3_OD) δ = 0.99 (tt, *J* = 10.2, 6.3 Hz, 2H), 1.16–1.30 (m, 4H), 2.50–2.64 (m, 2H), 2.75–3.03 (m, 10H), 3.44 (dd, *J* = 7.8, 5.4 Hz, 2H), 4.45 (t, *J* = 7.4 Hz, 2H), and 7.21–7.01 (m, 20H); ^13^C{^1^H} NMR (100 MHz, CD_3_OD) *δ* = 25.0, 29.8, 39.4, 40.2, 42.0, 55.8, 57.4, 127.8, 127.9, 129.5, 129.6, 130.5, 138.2, 138.8, 172.9, and 176.5; HRMS (ESI/Q-TOF) m/z: [M + H]^+^ calculated for C_41_H_50_N_6_O_4_ 691.3972; found 691.3973.

##### Synthesis of **6d**

The compound **6d** was successfully synthesized following the same procedure as described for **6a**, but using **5d** instead of **5a**. The yield was 0.485 g (0.662 mmol, 70.1%); the mp was 138–141 °C; [α]_D_^25^ = −3.02° (*c* = 0.05, DMSO); ATR-FTIR: 3272, 3085, 1654, and 1542 cm^−1^; ^1^H NMR (400 MHz, CD_3_OD) δ = 1.01–1.18 (m, 10H), 1.26 (p, *J* = 7.7, 6.9 Hz, 4H), 2.51–2.65 (m, 2H), 2.76–3.03 (m, 10H), 3.44 (dd, *J* = 7.7, 5.5 Hz, 2H), 4.45 (t, *J* = 7.3 Hz, 2H), and 7.20–7.01 (m, 10H); ^13^C{^1^H} NMR (100 MHz, CD_3_OD) *δ* = 27.8, 30.2, 39.4, 40.4, 42.1, 55.8, 57.4, 127.8, 127.9, 129.5, 129.6, 130.5, 138.2, 138.8, 172.3, and 176.5; HRMS (ESI/Q-TOF) m/z: [M + H]^+^ calculated for C_44_H_56_N_6_O_4_ 733.4441; found 733.4449.

##### Synthesis of **6e**

The compound **6e** was successfully synthesized following the same procedure as described for **6a**, but using **5e** instead of **5a**. The yield was 0.263 g (0.475 mmol, 77.4%); the mp was 207–209 °C; [α]_D_^25^ = −1.75° (*c* = 0.05, DMSO); ATR-FTIR: 3280, 3085, 1636, and 1546 cm^−1^; ^1^H NMR (400 MHz, DMSO-*d*_6_) δ = 0.80 (dd, *J* = 6.8, 5.0 Hz, 6H), 1.92 (h, *J* = 6.6 Hz, 2H), 2.63 (dd, *J* = 13.4, 8.5 Hz, 2H), 2.99 (dd, *J* = 13.5, 4.3 Hz, 2H), 3.11 (s, 4H), 3.46 (dd, *J* = 8.6, 4.3 Hz, 2H), 4.10 (t, *J* = 7.3 Hz, 2H), 7.38–7.14 (m, 10H), 7.96 (d, *J* = 9.0 Hz, 2H), and 8.03 (s, 2H); ^13^C{^1^H} NMR (100 MHz, DMSO-*d*_6_) *δ* = 18.5, 19.6, 31.3, 41.1, 56.5, 57.7, 126.6, 128.6, 129.8, 139.1, 171.4, and 174.4; HRMS (ESI/Q-TOF) m/z: [M + H]^+^ calculated. for C_30_H_44_N_6_O_4_ 553.3497; found 553.3503.

##### Synthesis of **6f**

The compound **6f** was successfully synthesized following the same procedure as described for **6a**, but using **5f** instead of **5a**. The yield was 0.428 g (0.774 mmol, 63.5%); the mp was 184–189 °C; [α]_D_^25^ = −8.2° (*c* = 0.05, DMSO); ATR-FTIR: 3282, 3083, 1636, and 1535 cm^−1^; ^1^H NMR (300 MHz, DMSO-*d*_6_) δ = 0.56 (dd, *J* = 6.8, 1.5 Hz, 6H), 0.73 (d, *J* = 6.9 Hz, 6H), 1.81 (tdd, *J* = 6.8, 4.7, 2.0 Hz, 2H), 2.58 (dd, *J* = 13.3, 8.3 Hz, 2H), 2.76–2.88 (m, 2H), 2.91–2.97 (m, 4H), 3.04 (d, *J* = 3.2 Hz, 2H), 4.43–4.52 (m, 2H), 7.18–7.27 (m, 10H), 7.90 (d, *J* = 5.9 Hz, 2H), and 8.05 (s, 2H); ^13^C{^1^H} NMR (75 MHz, DMSO-*d*_6_) *δ* = 17.0, 17.1, 19.9, 31.5, 38.5, 126.7, 128.5, 129.6, 138.2, 171.6, and 174.7; HRMS (ESI/Q-TOF) m/z: [M + H]^+^ calculated for C_30_H_44_N_6_O_4_ 553.3497; found 553.3508.

## Figures and Tables

**Table 1:** Gelation properties of compounds **6a**–**f** in aqueous solutions ^1^.

Entry	Comp.	Solvent(s)	Proportion (*v*/*v*)	Conc. (mg/mL)	Result
1	**6a**	H_2_O:DMSO	90:10	1	S
2	**6b**	H_2_O:DMSO	90:10	1	G ^4^
3	**6c**	H_2_O:DMSO	90:10	1	wG ^4^
4	**6c**	H_2_O:DMSO	90:10	3	G ^4^
5	**6d**	H_2_O:DMSO	90:10	1	wG ^2^
6	**6d**	H_2_O:DMSO	90:10	5	wG ^3^
7	**6e**	H_2_O:DMSO	90:10	1	S
8	**6e**	H_2_O:DMSO	90:10	5	S
9	**6f**	H_2_O:DMSO	90:10	1	S
10	**6f**	H_2_O:DMSO	90:10	5	wG ^3^
11	**6e** + **6f**	H_2_O:DMSO	90:10	0.5 + 0.5	wG
12	**6b**	H_2_O:DMSO	50:50	1	wG ^3^
13	**6a**	H_2_O:EtOH	90:10	1	I
14	**6b**	H_2_O:EtOH	90:10	1	I
15	**6b**	H_2_O:EtOH	70:30	1	wG ^3^
16	**6b**	H_2_O:EtOH	70:30	0.5	wG ^3^
17	**6b**	H_2_O:EtOH	50:50	1	G ^3^
18	**6a**	H_2_O:EtOH	50:50	1	S
19	**6a**	H_2_O	-	1	I ^2^
20	**6b**	H_2_O	-	1	G ^2^

^1^ The gel formation was qualitatively analysed by the vial inversion technique. I: insoluble, S: soluble, wG: weak gel, and G: gel. ^2^ Heating at 90 °C for 5 min. ^3^ Ultrasonication for 5 min. ^4^ Instantaneous formation of gel.

**Table 2:** Gelation properties as a function of pH for **6b** (1 mg/mL) in H_2_O:DMSO (90:10 *v*/*v*).

Vial	Buffer (mM)	pH	Result ^1^
1	- ^2^	1.2	S
2	Citrate (20)	4.4	wG
3	Citrate (20)	6.0	wG
4	Phosphate (30)	7.1	wG
5	HEPES (20)	8.1	G

^1^ Gel formation was qualitatively analysed by the vial inversion technique. ^2^ The sample at pH = 1 was prepared by adding 20 μL of concentrated HCl. S: soluble, wG: weak gel, and G: gel. The buffers were commercially available and, when needed, the pH was adjusted using NaOH or HCl. See Figure S3 for the results of the vial inversion tests.

## Data Availability

Not applicable.

## Supplementary Materials

The following supporting information can be downloaded at: https://www.mdpi.com/article/10.3390/gels8060390/s1: Table S1: CGC results by vial inversion; Figure S1: Vial inversion images for determining CGC; Table S2: Thermal stability results using vial inversion; Figure S2: Rheological measurements for **6b** (5 mg/mL); Figure S3: pH dependence vial inversion tests; Figure S4: SEM images for (**6b**·2HCl) crystals; Figure S5: Partial ^1^H NMR spectra for **6b** titration with H_2_O; Figure S6: Partial ^1^H NMR spectra for **6b** and **6f** titrations with H_2_O; Figure S7: Partial ^1^H NMR spectra (aromatic region) for **6b** and **6f** titrations with H_2_O; Figure S8: Variable temperature ^1^H NMR spectra; Figure S9: MMFFaq non-covalent forces **6b**-dimer; Figure S10: Partial ^1^H NMR spectra for CO_2_ absorption with **6b**; Figure S11: Time evolution ^1^H NMR spectra for CO_2_ absorption with **6b**; Figure S12: Main approaches for carbamate formation with diamines; Figure S13: Molecular models for ammonium carbamate **6b**-CO_2_ species; spectroscopic characterization, cartesian coordinates.
