# Supplementary material for: Unravelling the Supramolecular Driving Forces in the Formation of CO2-Responsive Pseudopeptidic Low-Molecular-Weight Hydrogelators
Source: Gels. 2022 Jun 20;8(6):390. doi: 10.3390/gels8060390 (PMC9222431; doi:10.3390/gels8060390)
Supplement: Supplementary file 1 [file gels-08-00390-s001.zip › gels-1777697-supplementary.pdf]

SUPPORTING INFORMATION

Unravelling the supramolecular driving forces in the formation of CO_2-responsive pseudopeptidic low molecular weight hydrogelators

Ferran Esteve[1], Alexis Villanueva-Antolí[2], Belen Altava[1,*], Eduardo García-Verdugo[1] and Santiago V. Luis[1,*]

[1] Departamento de Química Inorgánica y Orgánica, Universitat Jaume I, Av. Sos Baynat s/n, 12071, Castellón, Spain

[2] Institute of Advanced Materials (INAM), Universitat Jaume I, 12071 Castelló de la Plana, Castellón, Spain

Corresponding Authors: * E-mail: altava@uji.es.* E-mail: luiss@uji.es.

Table of contents

Table S1. CGC results by vial inversion S1
Figure S1. Vial inversion images for determining CGC S1
Table S2. Thermal stability results using vial inversion S2
Figure S2. Rheological measurements for **6b** (5 mg/mL) S2
Figure S3. pH dependence vial inversion tests S3
Figure S4. SEM images for [**6b**·2HCl] crystals S3
Figure S5. Partial ^{1}H NMR spectra for **6b** titration with H_2O S4
Figure S6. Partial ^{1}H NMR spectra for **6b** and **6f** titrations with H_2O S4
Figure S7. Partial ^{1}H NMR spectra (aromatic region) for **6b** and **6f** titrations with H_2O S5
Figure S8. Variable temperature ^{1}H NMR spectra S5
Figure S9. MMFFaq non-covalent forces **6b**-dimer S6
Figure S10. Partial ^{1}H NMR spectra for CO_2 absorption with **6b** S6
Figure S11. Time evolution ^{1}H NMR spectra for CO_2 absorption with **6b** S7
Figure S12. Main approaches for carbamate formation with diamines S7
Figure S13. Molecular models for ammonium carbamate **6b**-CO_2 species S8

Spectroscopic characterisation S9

Molecular modelling
Computational data S15

Table S1. Gelation properties for compound **6b** in DMSO:H_2O (10:90) at different concentrations.[a]

Entry	w/v (mg/mL)	mM	Result [a]
1	1.00	1.55	G
2	0.80	1.23	wG
3	0.60	0.91	wG
4	0.30	0.48	S
5	0.15	0.24	S
6	0.05	0.07	S

[a] Vial inversion method used for qualitative analyses. S: Soluble, wG: Weak Gel, G: Gel.

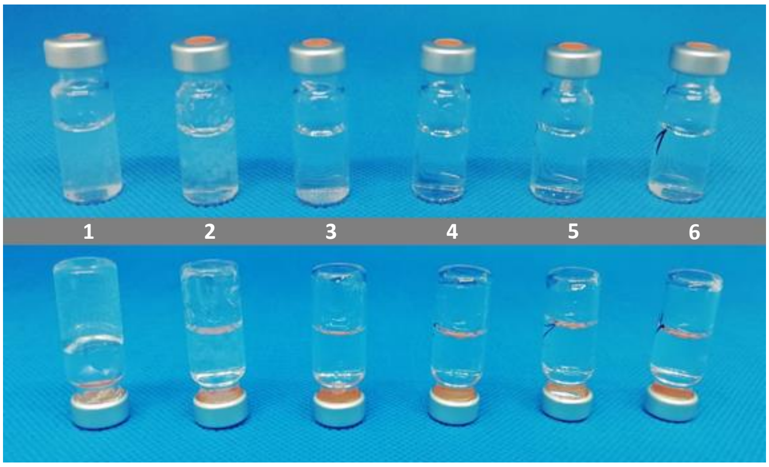

Figure S1. Vial inversion test pictures for determining the CGC of **6b** in H_2O:DMSO (90:10). The white numbers correspond to their assigned entry in Table 2.

Table S2. Thermal stability results for the hydrogel of **6b** (1 mg/mL in H_2O:DMSO 90:10).[a]

Temperature (ºC)	Result
25	G
30	G
35	G
40	G
45	G
50	G
55	G
60	G
65	G
70	G
75	G
80	G
85	G
90	wG

[a] The gel formation was qualitatively analysed by the vial inversion technique. I: Insoluble, S: Soluble, wG: Weak Gel, G: Gel. The sample was heated using an oil bath on a heating mantle.

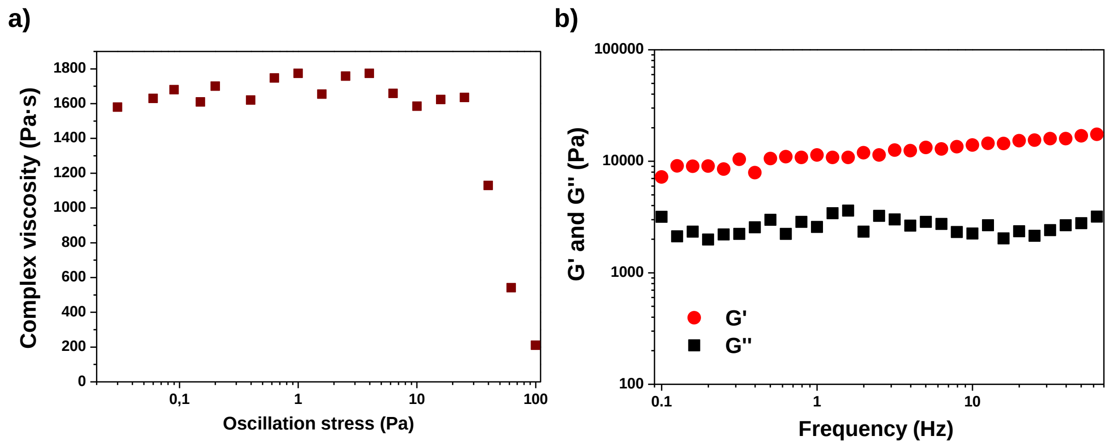

Figure S2. Rheological measurements (amplitude sweeps) for the hydrogel from **6b** (5 mg/mL, H_2O:DMSO (90:10 v/v). (a) The complex viscosity has been represented *vs* the oscillation stress. The frequency was set to 1 Hz. (b) Frequency sweeps with 0.1 Pa strain at 25 ºC.

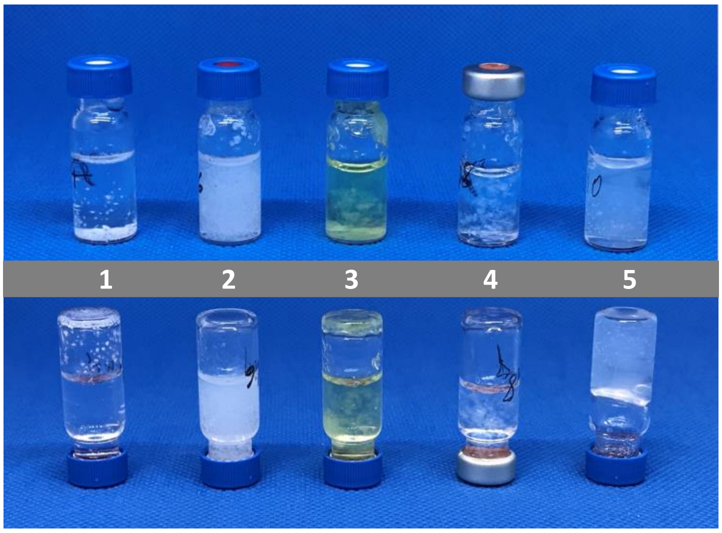

Figure S3. Effect of the pH in the gel formation for **6b** (1 mg/mL, bufferH_2O:DMSO 90:10). Vial pictures at pH: 1 (vial 1), 6 (vial 2), 7 (vial 3), 4 (vial 4), and 8 (vial 5). The vial at pH = 7 has a yellowish colour because the buffer used (Aldrich) presented a yellow colour for easy recognition.

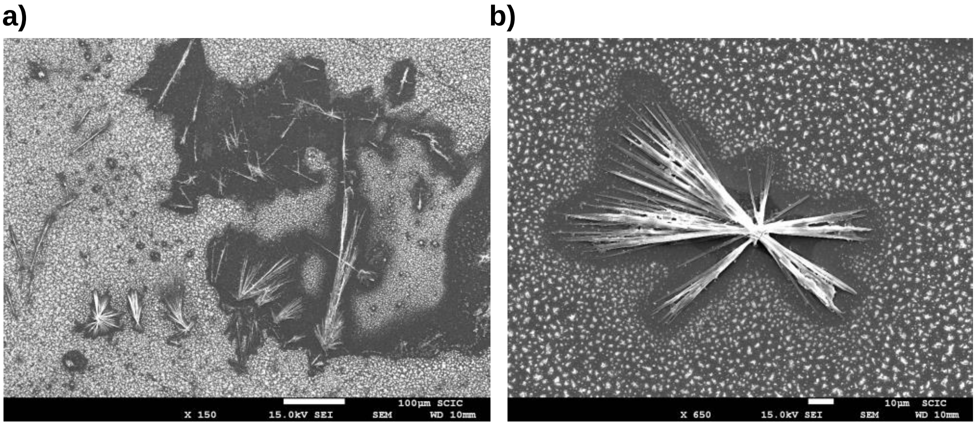

Figure S4. SEM images for the crystals obtained for a dried sample of **6b** (1 mg/mL) in DMSO:H_2O (10:90) at pH = 1. The crystalline solid has been assigned to the diprotonated [**6b**·2HCl] species.

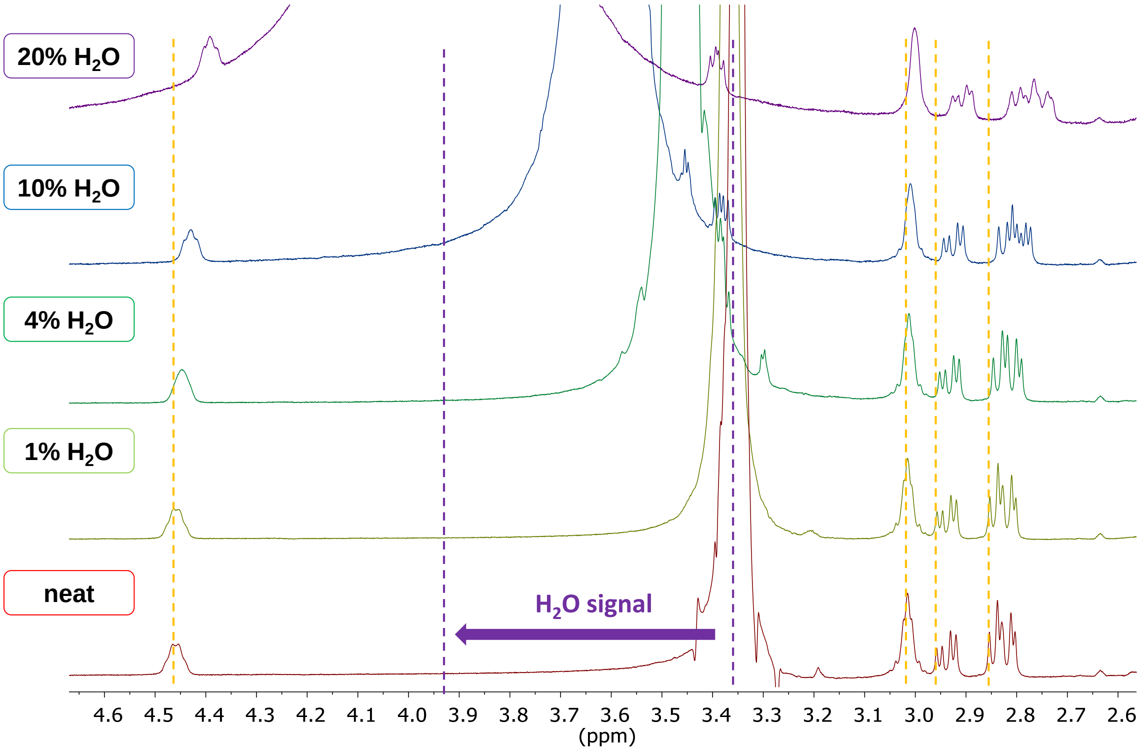

Figure S5. Partial ¹H NMR (500 MHz, DMSO-d_6) spectra for the titration of **6b** (3 mM) with increasing amounts of water. The water content has been indicated as v/v (%).

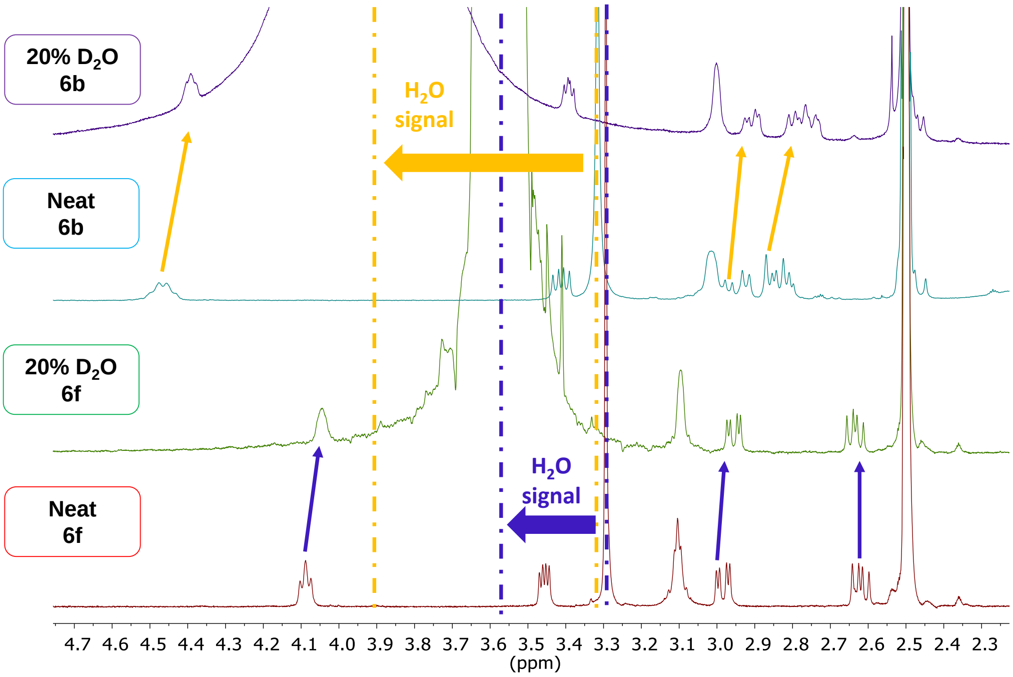

Figure S6. Partial ¹H NMR (500 MHz, DMSO-d_6, 3 mM) spectra for **6b** (above) and **6f** (below) in the presence and absence of H_2O. The spectra display the 4.7 – 2.3 ppm region. The water content has been indicated as v/v (%). The most relevant shifts have been highlighted in blue and orange for **6f** and **6b**, respectively.

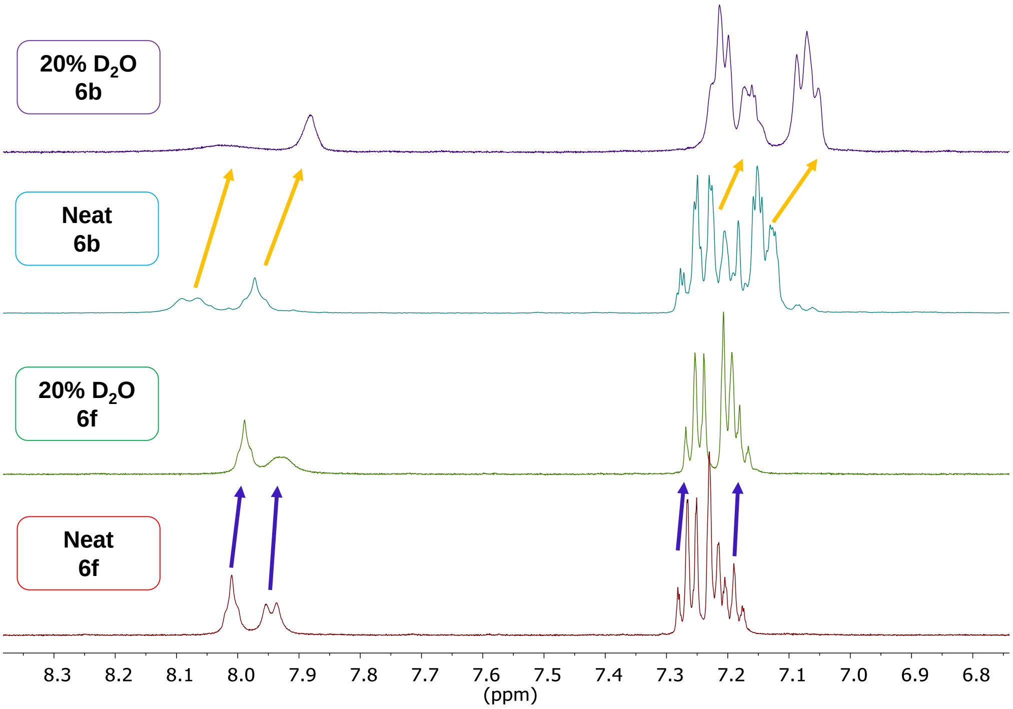

Figure S7. Partial ¹H NMR (500 MHz, DMSO-d_6, 3 mM) spectra for **6b** (above) and **6f** (below) in the presence and absence of H_2O. The spectra display the 8.3 – 6.8 ppm region. The water content has been indicated as v/v (%). The most relevant shifts have been highlighted in blue and orange for **6f** and **6b**, respectively.

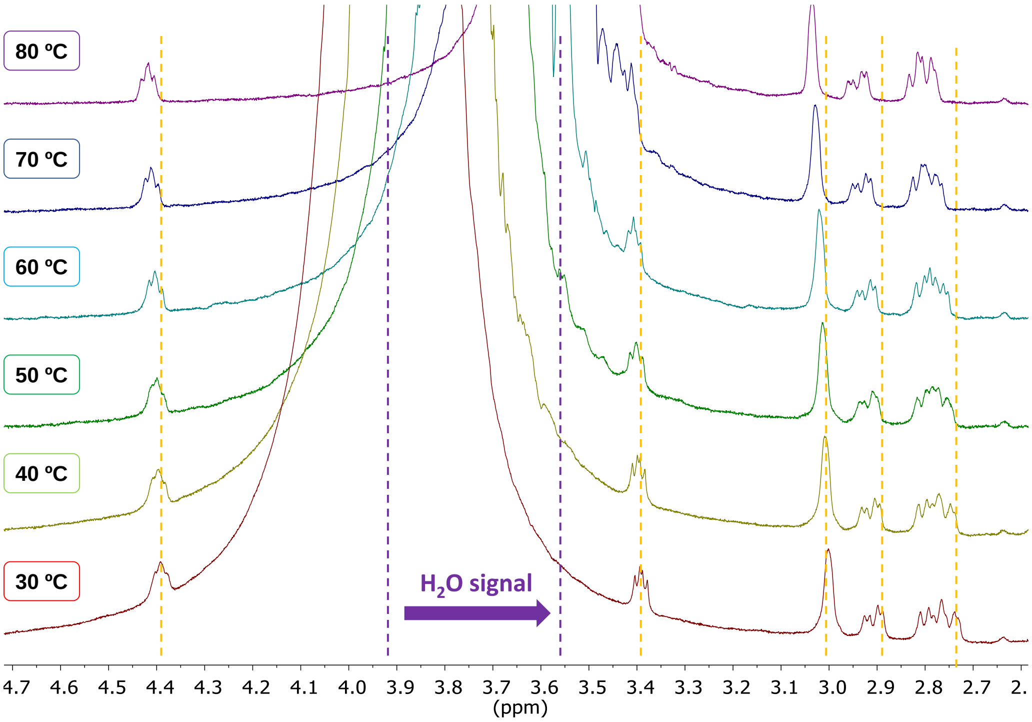

Figure S8. Partial ¹H NMR (500 MHz, DMSO-d_6) spectra for the variable temperature experiments of **6b** (3 mM, H_2O : DMSO, 20 : 80). The spectra display the 4.7 – 2.6 ppm region. Water shift has been highlighted in purple. Shifts for the characteristic protons of **6b** are highlighted in orange.

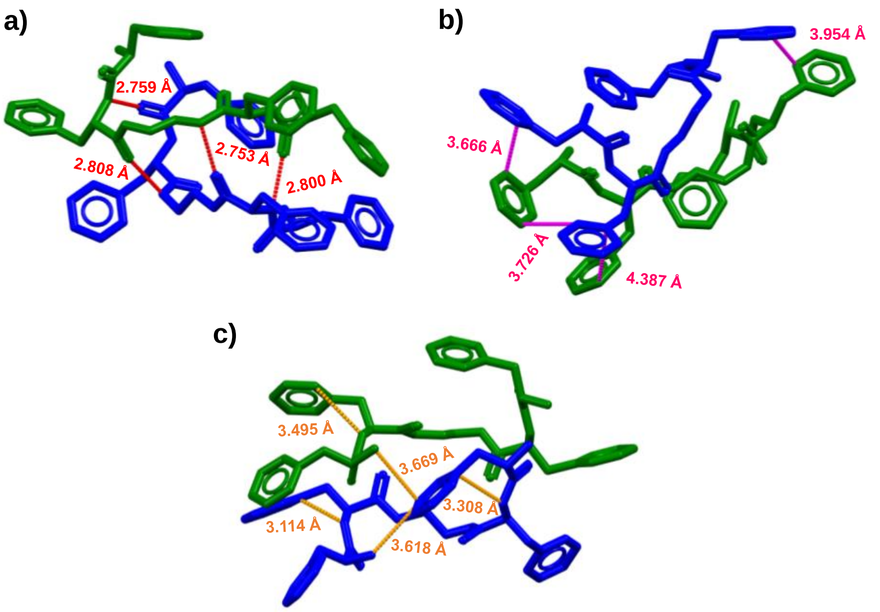

Figure S9. Non-covalent forces identified in the most stable conformation obtained for the dimer of **6b** (Spartan08', MMFFaq). (a) Intermolecular hydrogen bonding between the amide groups of the two different molecules. Bonds and distances have been highlighted in red. Measured distances correspond to O···N bonds in the C=O···N-H entities. (b) Intermolecular edge-to-face π-π interactions between the dimeric molecules. Bonds and distances have been highlighted in pink. Measured distances correspond to the closest aromatic carbons between the vicinal rings. (c) Intramolecular NH··· π interactions between the aromatic rings and the acidic NH protons of the amide groups. Bonds and distances have been highlighted in orange. Measured distances correspond to the closest aromatic carbon to the N atom of the amides.

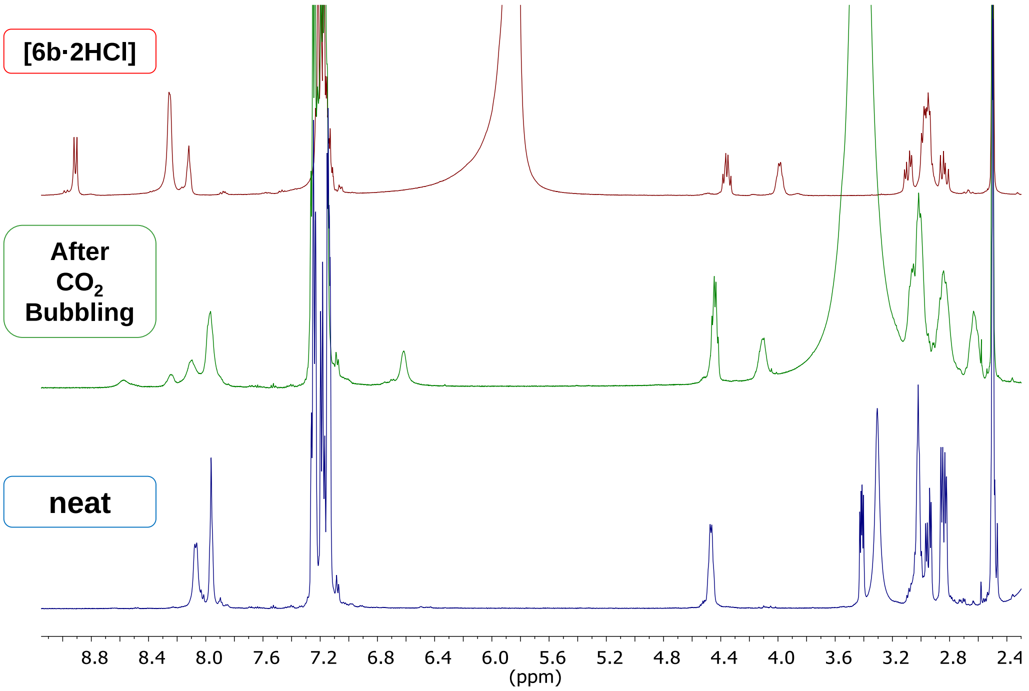

Figure S10. Partial ^1H NMR (500 MHz, 35 mM in **6b**, DMSO-d_6) spectra for neat **6b** (blue spectrum), **6b** sample after CO_2 bubbling (green spectrum), and [**6b**·2HCl] (red spectrum).

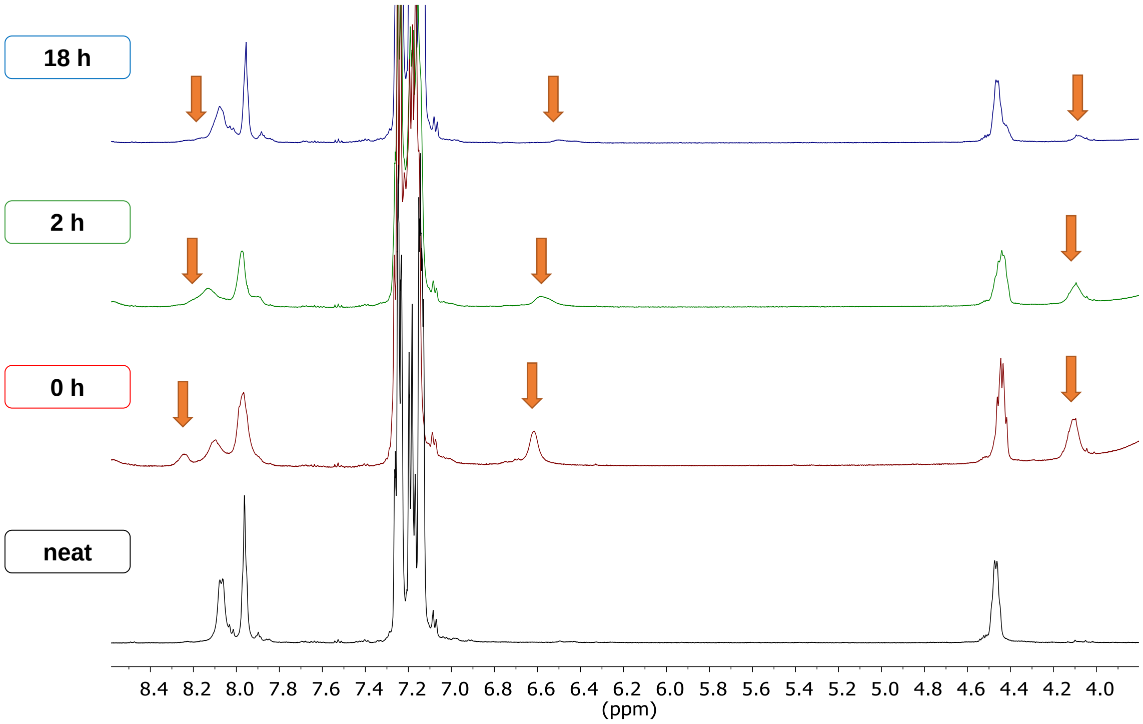

Figure S11. Time evolution of the partial ^{1}H NMR (500 MHz, 35 mM in **6b**, 25 ºC, DMSO-d_6) spectra for **6b** at different times after bubbling CO_2. The signals highlighted with orange arrows have been assigned to the metastable carbamate-derived species.

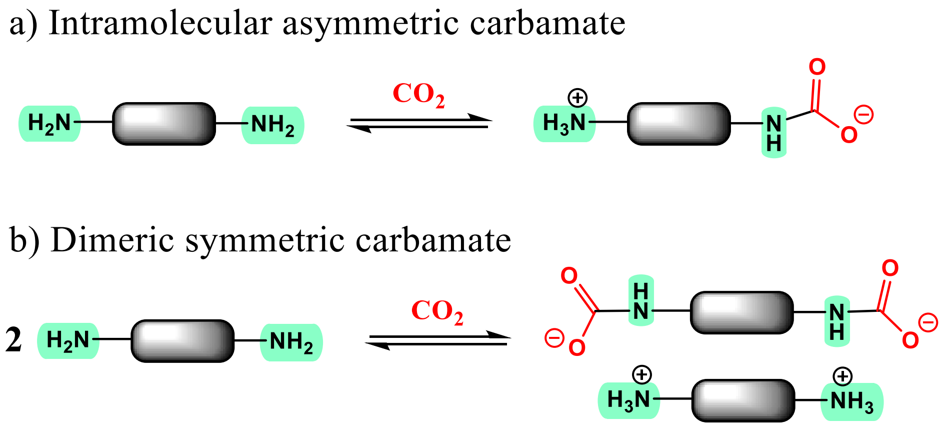

Figure S12. Main approaches described for the CO_2 absorption using diamino compounds. (a) Intramolecular asymmetric ammonium carbamate formation. (b) dimeric symmetric ammonium carbamate formation.

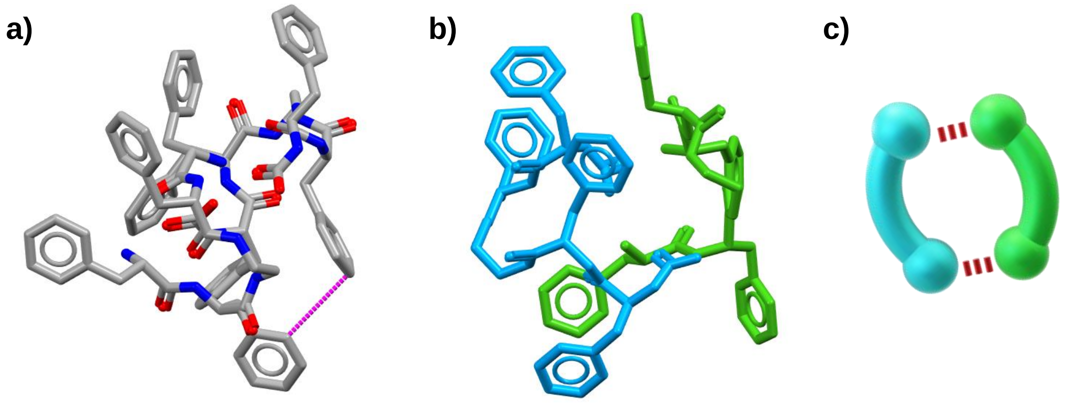

Figure S13. a) Most stable conformation obtained for the **6b**-CO_2 ammonium carbamate-derived symmetric dimer (Spartan08', MMFFaq). The presence of only one π-π interaction has been highlighted with a discontinuous magenta line. b) Most stable conformation obtained for the **6b**-CO_2 ammonium carbamate-derived symmetric dimer, with each one pseudopeptidic molecule coloured in green and the other one in light blue (Spartan08', MMFFaq). c) Representation of the supramolecular macrocyclic conformation adopted by the dimeric ammonium carbamate derivative. Electrostatic interactions have been highlighted with discontinuous red lines.

SPECTROSCOPIC CHARACTERISATION

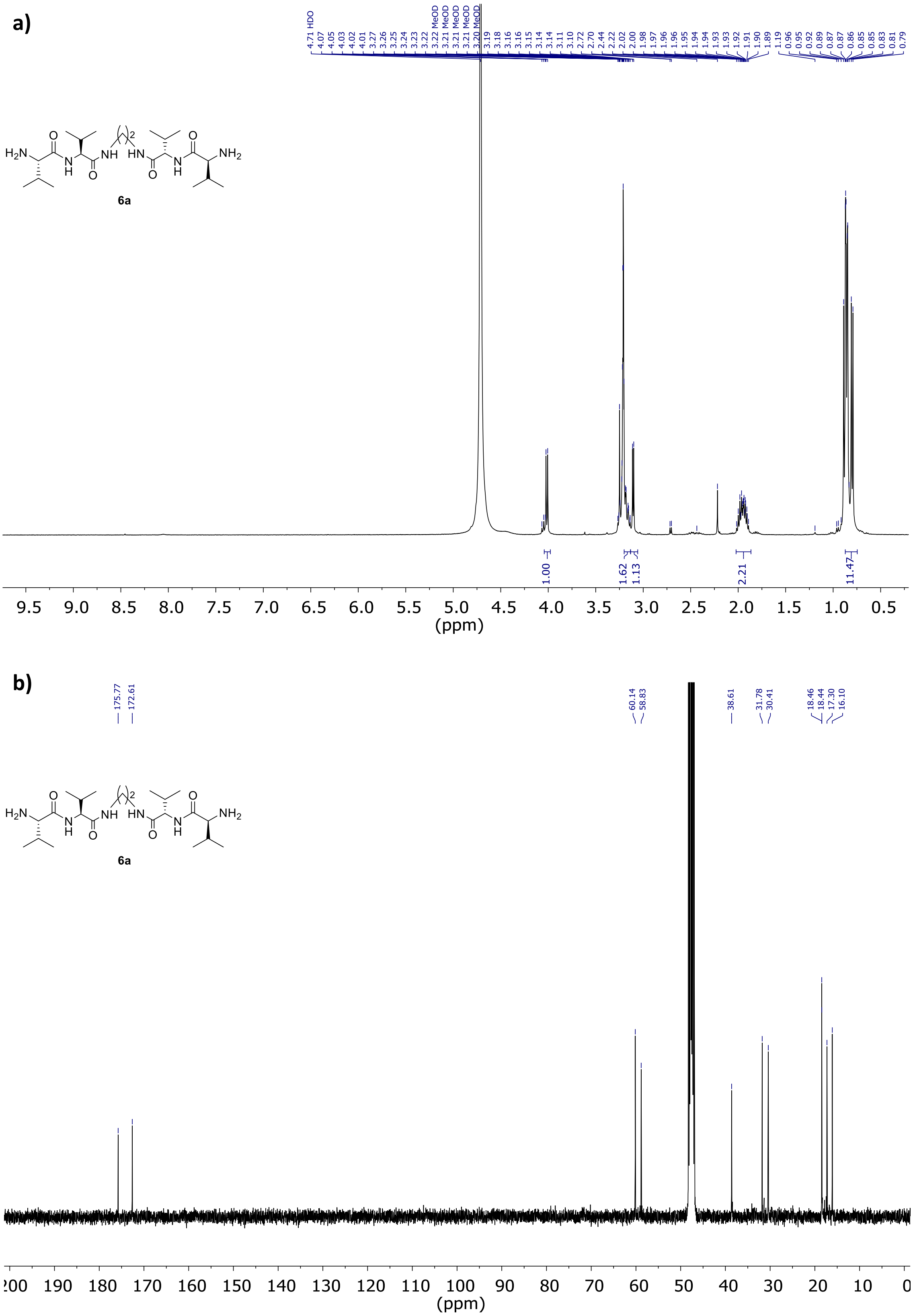

Figure S14. a) ^{1}H NMR (400 MHz, CD_3OD) for **6a** (7 mM). b) $^{13}C\{^{1}H\}$ NMR (75 MHz, CD_3OD) for **6a** (10 mM).

a)

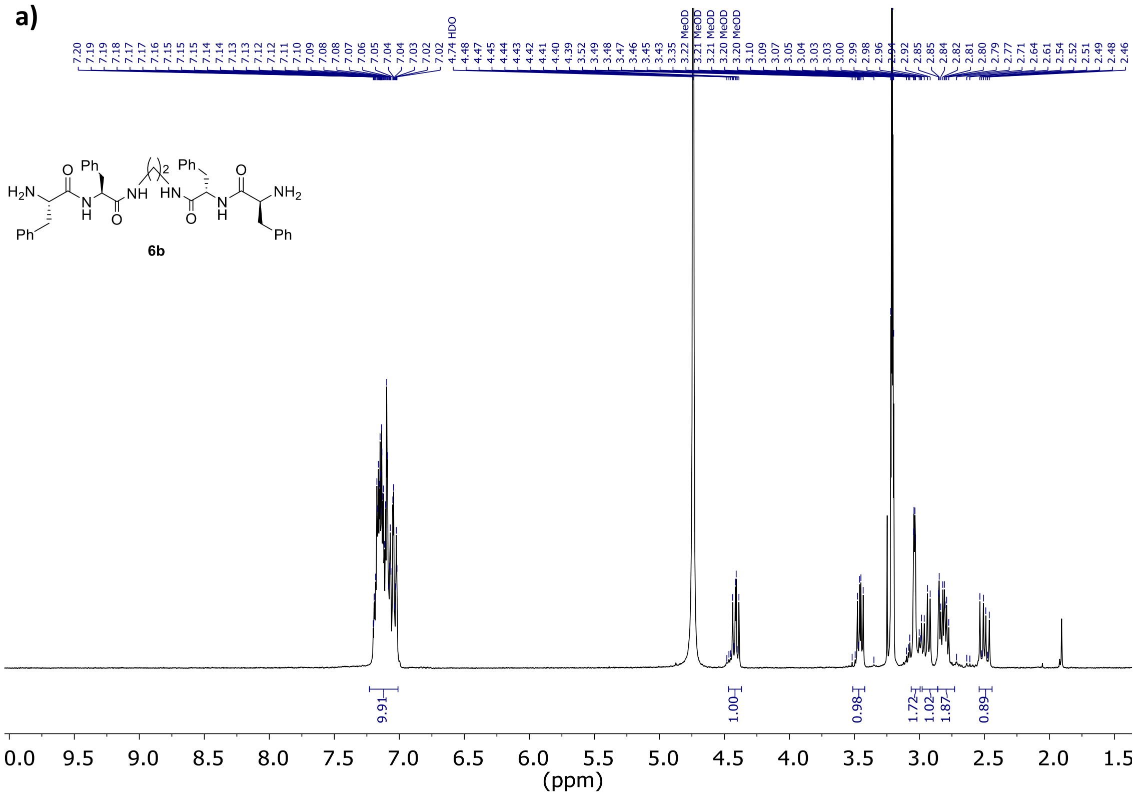

b)

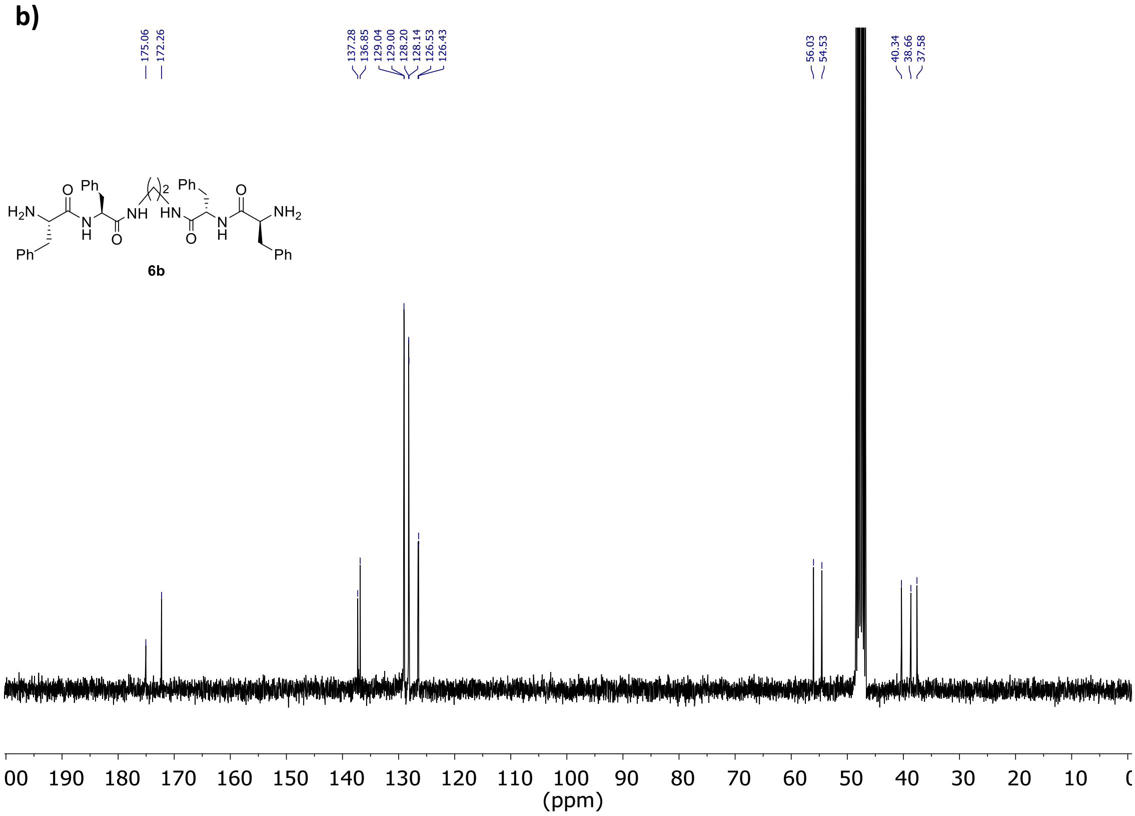

Figure S15. a) ^{1}H NMR (300 MHz, CD_3OD) for **6b** (7 mM). b) $^{13}C\{^{1}H\}$ NMR (75 MHz, CD_3OD) for **6b** (7 mM).

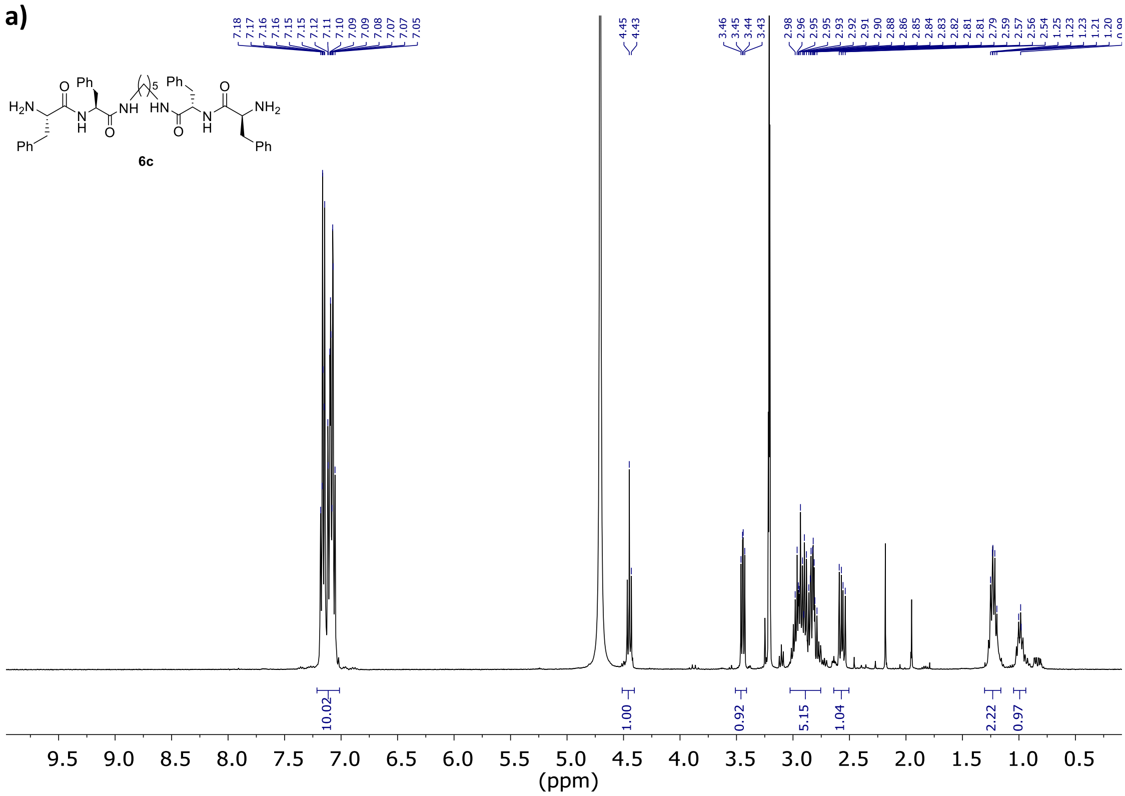

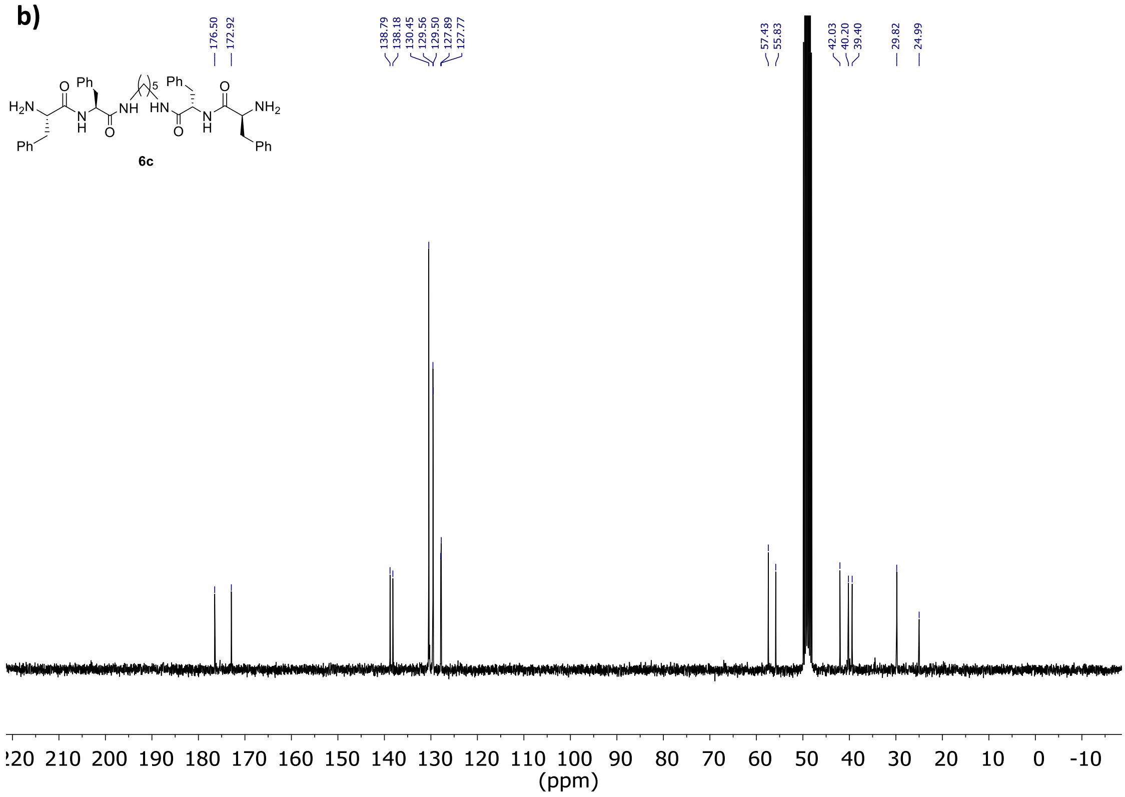

Figure S16. a) ^{1}H NMR (400 MHz, CD_3OD) for **6c** (10 mM). b) $^{13}C\{^{1}H\}$ NMR (100 MHz, CD_3OD) for **6c** (10 mM).

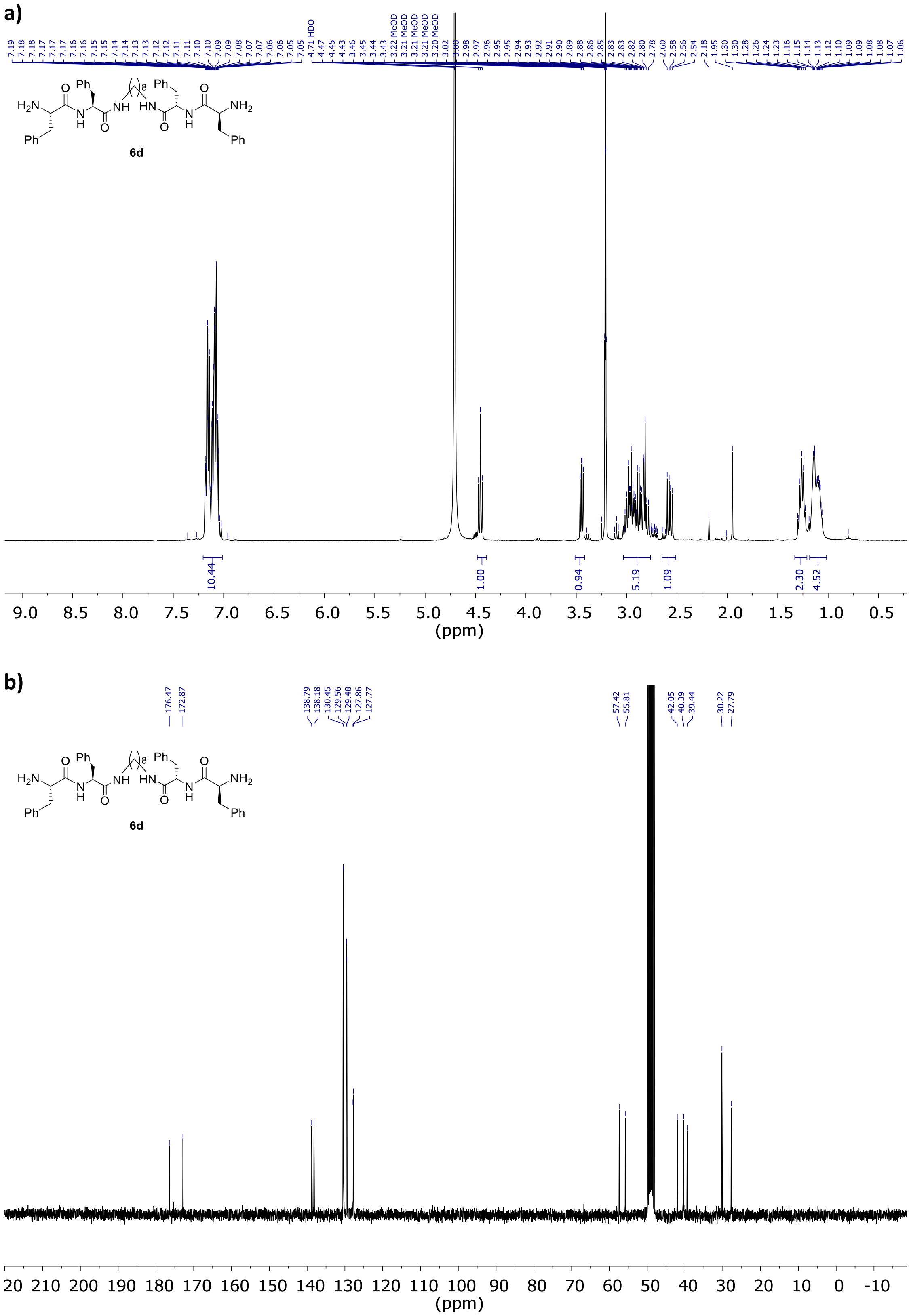

Figure S17. a) ^{1}H NMR (400 MHz, CD_3OD) for **6d** (7 mM). b) $^{13}C\{^{1}H\}$ NMR (100 MHz, CD_3OD) for **6d** (7 mM).

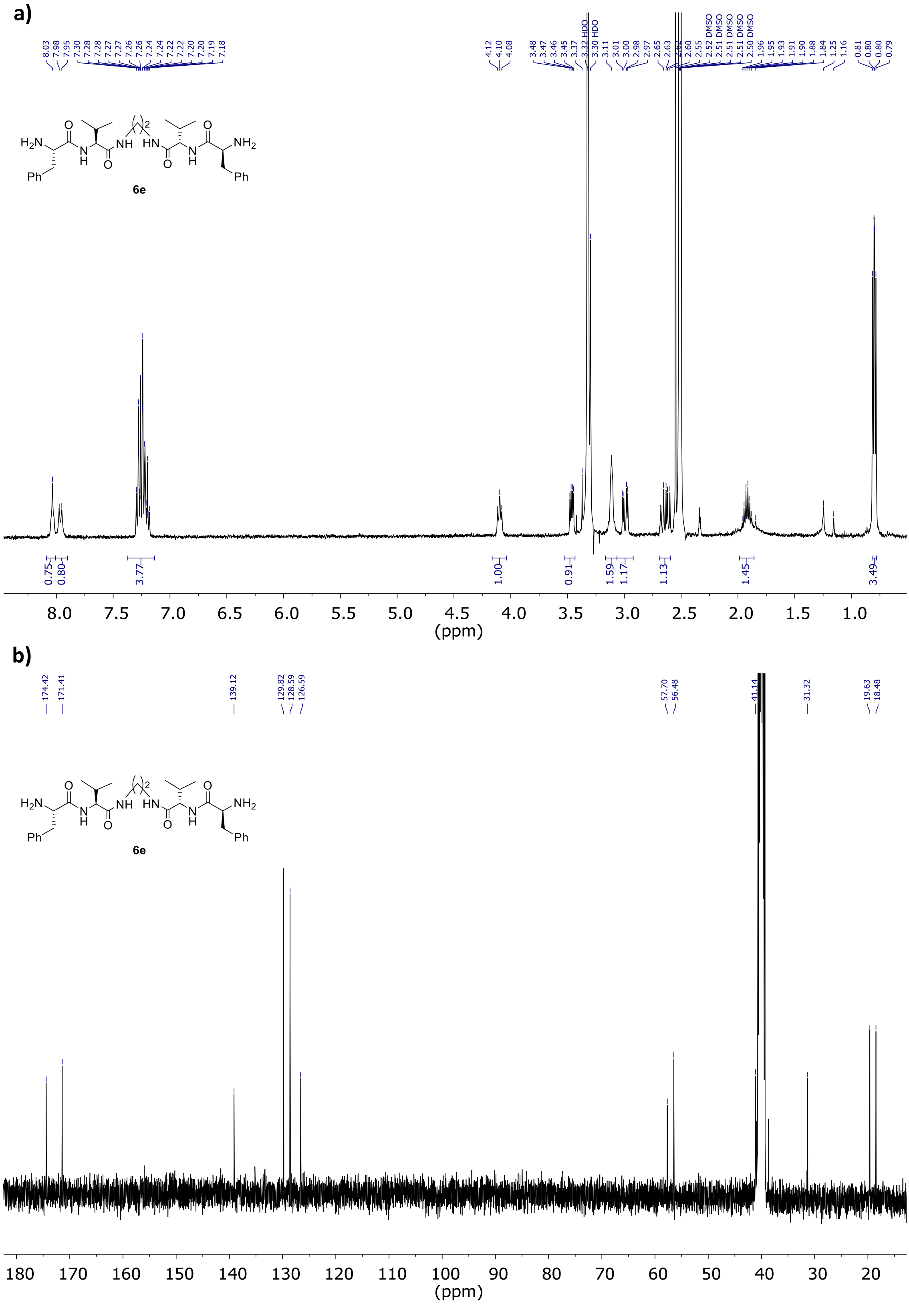

Figure S18. a) ^{1}H NMR (400 MHz, DMSO-d_6) for **6e** (4 mM). b) $^{13}C\{^{1}H\}$ NMR (100 MHz, CD_3OD) for **6e** (4 mM).

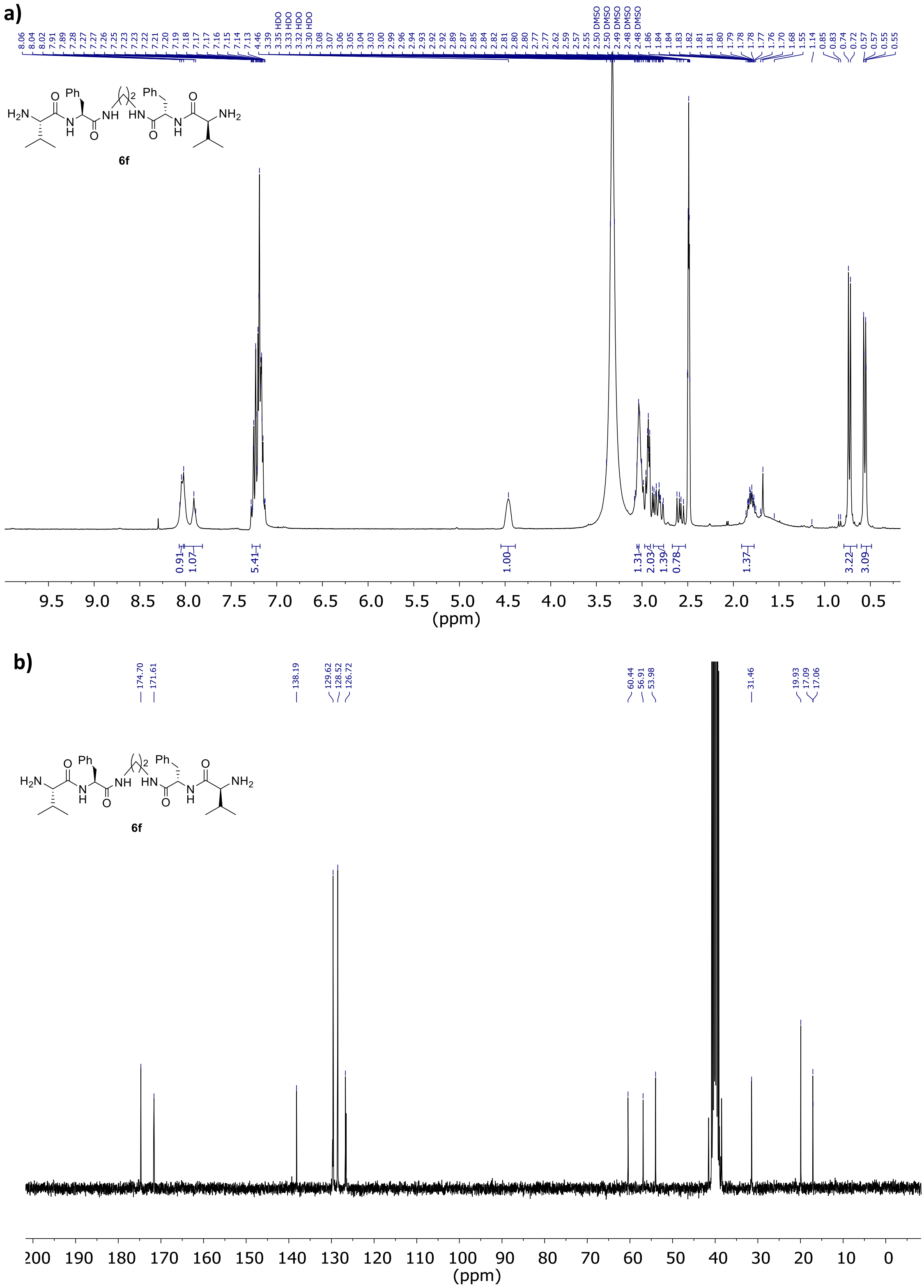

Figure S19. a) ^{1}H NMR (300 MHz, DMSO-d_6) for **6f** (10 mM). b) $^{13}C\{^{1}H\}$ NMR (75 MHz, CD_3OD) for **6f** (10 mM).

MOLECULAR MODELLING

- Lowest energy conformation for [**6b·6b**] dimer.

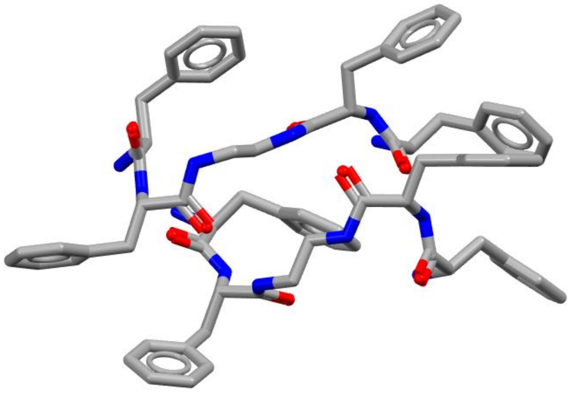

Cartesian coordinates (184 atoms), E (298 K) = 860.24 kJ/mol; Eaq (298 K) = 486.59 kJ/mol

C	1	-0.07424534	-0.70290665	2.52862009
H	2	0.42555888	0.25048081	2.72392708
H	3	-0.43413776	-0.66821846	1.49416133
C	4	-1.23625109	-0.88273169	3.50398312
H	5	-0.89026588	-0.76186228	4.53581973
H	6	-1.66471707	-1.88710137	3.40977392
N	7	-2.27745557	0.10018256	3.27288543
H	8	-2.06146754	1.06160334	3.54952506
N	9	0.90257573	-1.76550368	2.59635581
H	10	0.56138026	-2.72304789	2.68284202
C	11	-3.20821234	-0.08113059	2.27258658
C	12	2.13415966	-1.62390144	1.98277367
O	13	-3.30078587	-1.1250605	1.62692378
O	14	2.59800623	-0.52225924	1.68497224
C	15	-4.14890401	1.10953571	2.02891573
H	16	-3.68967282	2.02193944	2.41627394
C	17	2.87720555	-2.96000158	1.75832052
H	18	2.98318974	-3.43962939	2.73992198
N	19	-4.30274993	1.24982752	0.57730809
N	20	2.05514457	-3.81374134	0.89043319
H	21	1.91071321	-3.47744459	-0.0680477
C	22	4.26451923	-2.71333976	1.14862054
H	23	4.77375827	-1.8968903	1.67719055
C	24	-5.51138583	0.85493271	2.69178078
C	25	1.11774621	-4.68024305	1.39193268
H	26	-4.31141874	0.38628457	0.03645441
C	27	-4.2416003	2.46304646	-0.06307797
O	28	0.85846082	-4.72259618	2.59667652
O	29	-4.06775302	3.52098257	0.54791214
C	30	-4.43450037	2.40808469	-1.5997795
H	31	-5.24121912	1.69713779	-1.81429069
C	32	0.30681593	-5.4967643	0.36847324
H	33	-0.08863626	-6.36102199	0.91864119
N	34	-4.89349899	3.73082592	-2.08379939

N	35	-0.83794017	-4.67388457	-0.08014855
C	36	-3.13118928	2.00118537	-2.31042899
H	37	-2.26725502	2.49341584	-1.84490888
C	38	1.1678062	-6.00593763	-0.8008552
H	39	-5.72663829	4.00047508	-1.5596162
H	40	-4.20154798	4.44596301	-1.82685011
H	41	-1.37438227	-4.39407044	0.74461305
H	42	-1.46560313	-5.27811458	-0.61162631
C	43	0.85856632	2.5539036	0.74625293
H	44	-0.07204614	1.98951218	0.87344839
H	45	0.65066967	3.34554278	0.01686933
C	46	1.30803246	3.17219532	2.07397366
H	47	2.27883928	3.66542355	1.95649904
H	48	1.41538811	2.39807828	2.84092676
N	49	0.37443167	4.17511193	2.54714216
H	50	0.46527129	5.11444065	2.15385347
N	51	1.84595082	1.65695139	0.18100958
H	52	2.28361953	0.98510557	0.82008625
C	53	-0.86475136	3.83994238	3.06367063
C	54	1.70435498	1.21231375	-1.11940894
O	55	-1.18517541	2.6864475	3.34876777
O	56	0.80394389	1.59841048	-1.86445314
C	57	-1.84150548	5.03377948	3.11944597
H	58	-1.2985125	5.91628309	3.47929558
C	59	2.817532	0.2607308	-1.59414314
H	60	3.06223637	-0.43026917	-0.78440521
N	61	-2.27179494	5.24715285	1.73423847
N	62	2.3107315	-0.50492421	-2.72691499
H	63	1.84498743	0.02392702	-3.4651268
C	64	4.04459475	1.10190653	-1.97520591
H	65	4.31476288	1.78144999	-1.15603306
C	66	-3.02144964	4.72226456	4.04530874
C	67	1.77783331	-1.76774176	-2.56866196
H	68	-2.90310373	4.55088672	1.3300356
C	69	-1.51268572	5.99828446	0.85764421
O	70	1.85343117	-2.42018076	-1.52948382
O	71	-0.52419945	6.62091387	1.23726144
C	72	-1.99572011	5.91625272	-0.60984401
H	73	-1.92552215	4.86547466	-0.91441742
C	74	1.09709395	-2.29209228	-3.85178833
H	75	0.42272125	-3.1097491	-3.57341248
N	76	-3.42733542	6.31019614	-0.672533
N	77	0.28977569	-1.18590226	-4.43813768
C	78	-1.15714839	6.79486104	-1.55308717
C	79	2.14400669	-2.74196682	-4.88330129
H	80	2.86381128	-1.93668845	-5.08434471
H	81	-3.93983935	5.76186923	0.02434569
H	82	-3.50654966	7.2778078	-0.35189504

H	83	-0.39301716	-0.87904474	-3.73551542
H	84	-0.27677107	-1.56241357	-5.19810901
H	85	4.17357225	-2.38058003	0.10749547
C	86	5.15224475	-3.93598642	1.19544548
C	87	6.76794079	-6.22495633	1.29218562
C	88	5.08931194	-4.89514854	0.17696334
C	89	6.03073815	-4.13953038	2.26752957
C	90	6.83639927	-5.27855427	2.31319637
C	91	5.89321767	-6.03412553	0.22482133
H	92	4.40496191	-4.75905194	-0.65784911
H	93	6.09345449	-3.41376373	3.07500751
H	94	7.51586925	-5.42925194	3.14795362
H	95	5.83315897	-6.77242163	-0.57003618
H	96	7.39375486	-7.11243077	1.33046959
H	97	1.4384575	-5.18477082	-1.47669875
H	98	2.12338478	-6.39232476	-0.42181699
C	99	0.4873391	-7.09602066	-1.59958937
C	100	-0.79616092	-9.11274608	-3.06324569
C	101	-0.323181	-6.77349507	-2.69679416
C	102	0.64253632	-8.44115689	-1.24067348
C	103	0.00423621	-9.44440791	-1.97130331
C	104	-0.96049313	-7.7774595	-3.42606864
H	105	-0.45542545	-5.73389585	-2.98885115
H	106	1.26397484	-8.71618687	-0.39121653
H	107	0.1315596	-10.4860016	-1.68782471
H	108	-1.58444049	-7.51875539	-4.27728273
H	109	-1.29209381	-9.89578613	-3.63077931
H	110	-6.28267556	1.46438863	2.20117291
H	111	-5.83055301	-0.18549859	2.5440549
C	112	-5.52604723	1.17404492	4.16808481
C	113	-5.49568269	1.80504045	6.90071563
C	114	-5.12279331	0.22158363	5.11264767
C	115	-5.92546509	2.44187361	4.61165305
C	116	-5.90626123	2.75714666	5.97071808
C	117	-5.1069497	0.53717942	6.47225235
H	118	-4.81350886	-0.77334406	4.79787446
H	119	-6.24988013	3.19856921	3.90078846
H	120	-6.20997883	3.74762471	6.30121552
H	121	-4.78968031	-0.20766531	7.19755238
H	122	-5.48149595	2.04986669	7.95939747
H	123	-3.14289572	2.34270782	-3.35464238
C	124	-2.92092704	0.50381312	-2.33393764
C	125	-2.59733569	-2.28002256	-2.32774181
C	126	-3.66958275	-0.30253215	-3.20324387
C	127	-1.99907556	-0.10135196	-1.47279194
C	128	-1.84206122	-1.48662086	-1.46699562
C	129	-3.50786251	-1.68843406	-3.19965034
H	130	-4.38748524	0.14743251	-3.88665718

H	131	-1.3986506	0.4998996	-0.79409524
H	132	-1.13091687	-1.9482967	-0.78527935
H	133	-4.09798145	-2.30628558	-3.87130389
H	134	-2.47365626	-3.35935132	-2.31223659
H	135	1.65494228	-2.94133053	-5.84700106
C	136	2.8976559	-3.98813765	-4.4842781
C	137	4.3090153	-6.30010256	-3.75698224
C	138	4.16317063	-3.89264032	-3.89145775
C	139	2.34500734	-5.25633847	-4.70228587
C	140	3.04626317	-6.40633383	-4.33556588
C	141	4.86912684	-5.04338658	-3.53970474
H	142	4.61045502	-2.91975168	-3.69880114
H	143	1.3644154	-5.36015533	-5.16025413
H	144	2.60341951	-7.38598923	-4.49846946
H	145	5.85338594	-4.9537623	-3.08681862
H	146	4.85446198	-7.19624486	-3.47497434
H	147	3.80564439	1.75352577	-2.82661401
C	148	5.25517091	0.25899454	-2.30613928
C	149	7.46664275	-1.35365837	-2.91566727
C	150	5.6386036	0.05299278	-3.63785671
C	151	5.99706901	-0.3479425	-1.28370198
C	152	7.09340081	-1.1558066	-1.58795085
C	153	6.74228634	-0.74656924	-3.93996456
H	154	5.07504477	0.50716175	-4.44972006
H	155	5.72085222	-0.2015821	-0.24197485
H	156	7.65421938	-1.63339561	-0.78832181
H	157	7.03147506	-0.90303005	-4.97594269
H	158	8.3208891	-1.982456	-3.15188986
H	159	-2.65296577	4.39256342	5.02577803
H	160	-3.60129333	3.88066056	3.65393581
C	161	-3.94665453	5.90063185	4.24132036
C	162	-5.64988807	8.09967447	4.58731679
C	163	-3.71290166	6.82422237	5.26822101
C	164	-5.04030164	6.09544021	3.38584089
C	165	-5.88846236	7.18928318	3.55982951
C	166	-4.56274198	7.9178256	5.44032811
H	167	-2.86859795	6.69856469	5.94224803
H	168	-5.23655038	5.39497609	2.57685328
H	169	-6.73455406	7.33260078	2.89291327
H	170	-4.37554455	8.62993471	6.23992233
H	171	-6.30980525	8.95260044	4.7223476
H	172	-1.04449988	7.80623828	-1.14081057
H	173	-1.67988785	6.92137555	-2.5112918
C	174	0.20408571	6.20836103	-1.86008037
C	175	2.72356296	5.10076385	-2.41261114
C	176	0.32684585	5.11658514	-2.72963125
C	177	1.36109125	6.73901156	-1.27397248
C	178	2.61369786	6.18678018	-1.54752269

C	179	1.58060258	4.56780074	-3.0041861
H	180	-0.5528371	4.67988815	-3.1979653
H	181	1.29195948	7.58309446	-0.59027071
H	182	3.50283133	6.60598597	-1.08439286
H	183	1.65848917	3.71318613	-3.67161864
H	184	3.697722	4.66998348	-2.62579609

- Lowest energy conformation for [**6e·6e**] dimer.

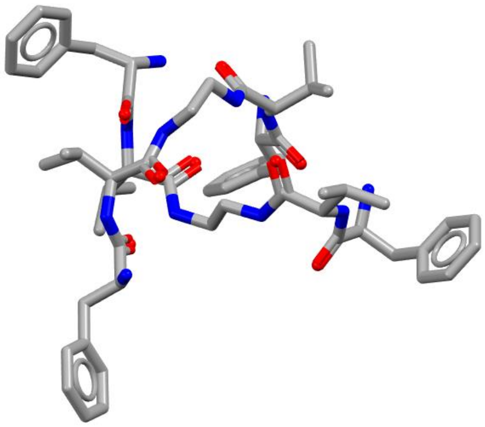

Cartesian coordinates (168 atoms), E (298 K) = 452.64 kJ/mol; Eaq (298 K) = 117.75 kJ/mol

C	1	-0.85398466	0.3525045	-3.96931102
H	2	-1.85742932	0.78239904	-3.88090829
H	3	-0.7663889	-0.08842552	-4.96823794
C	4	0.21848567	1.4326701	-3.8011462
H	5	0.19918763	2.10132535	-4.66922573
H	6	1.21981346	0.9911682	-3.7452146
N	7	0.00638247	2.26879059	-2.63579501
H	8	-0.95283043	2.40927791	-2.30364593
N	9	-0.69907653	-0.74600257	-3.03538241
H	10	0.25113852	-1.08893516	-2.86307165
C	11	0.97212977	3.14232691	-2.18896366
C	12	-1.75774021	-1.41176008	-2.46497359
O	13	2.13027773	3.13826877	-2.60696044
O	14	-2.92845981	-1.04732548	-2.59600301
C	15	0.47284225	4.16691857	-1.14300831
H	16	-0.16142291	3.61870958	-0.4407678
C	17	-1.37730053	-2.68863171	-1.67855927
H	18	-0.3982657	-2.53334844	-1.21218032
N	19	1.62284439	4.67788342	-0.40748657
N	20	-2.33897126	-2.84573055	-0.59776679
H	21	-3.27231428	-2.45576527	-0.70716014
C	22	-1.33061268	-3.96287794	-2.55488329
H	23	-1.217052	-4.8246428	-1.8826058
C	24	-2.61848236	-4.19239183	-3.35286094
H	25	-2.76748354	-3.41878219	-4.11307522
H	26	-2.58112464	-5.1580109	-3.86913842
H	27	-3.49564543	-4.20444051	-2.69814085
C	28	-0.12506014	-3.9833066	-3.4942148
H	29	0.80476651	-3.88125772	-2.92864163
H	30	-0.17091433	-3.18265909	-4.23823844
H	31	-0.07511432	-4.93453802	-4.03538862
C	32	-0.34966935	5.29347524	-1.80661609
H	33	-1.12729331	4.83780351	-2.43263368
C	34	0.48505474	6.19617634	-2.72077782

H	35	0.98154157	5.61681232	-3.50529135
H	36	1.25077918	6.74454662	-2.16248533
H	37	-0.15516513	6.93525943	-3.21503269
C	38	-1.05940914	6.13999391	-0.74559702
H	39	-0.34431166	6.65083896	-0.09222378
H	40	-1.7068064	5.51644736	-0.11993658
H	41	-1.68652617	6.90420892	-1.21636782
C	42	-2.03328287	-3.4078714	0.61736538
H	43	2.40841203	5.0107761	-0.96917051
C	44	2.03300859	4.1027591	0.77466633
O	45	-0.94576338	-3.92833377	0.85989805
O	46	1.32387921	3.3677977	1.45923475
C	47	3.45522796	4.51767668	1.19489654
H	48	3.90271909	3.66846485	1.72476685
C	49	-3.18969099	-3.3078696	1.63689252
H	50	-3.31823261	-2.24601418	1.873932
N	51	4.26845265	4.78411907	-0.02315374
N	52	-4.44496803	-3.78100277	0.99415235
C	53	3.35442388	5.75488408	2.10044141
H	54	2.59658918	5.58975224	2.87861304
C	55	-2.84053306	-4.08684798	2.91836368
H	56	-2.70128333	-5.15106906	2.68434075
H	57	5.21756132	5.01700852	0.27669175
H	58	4.35617029	3.9123573	-0.55301284
H	59	-4.36251945	-4.78249747	0.8217001
H	60	-5.20006951	-3.68051753	1.6752476
C	61	1.3423993	-0.05765376	0.09390364
H	62	1.83160207	0.88057954	-0.19023441
H	63	0.5064104	-0.20488317	-0.59472041
C	64	0.83540215	0.02499858	1.53553905
H	65	1.6631425	0.24476688	2.21829561
H	66	0.397094	-0.93270027	1.83921291
N	67	-0.1485379	1.07677352	1.69887821
H	68	0.21618651	2.03329078	1.71250464
N	69	2.31172822	-1.11513428	-0.09337392
H	70	2.87450732	-1.42748946	0.69932581
C	71	-1.46658213	0.87760836	1.35362564
C	72	2.52341509	-1.73459369	-1.30768908
O	73	-1.91579578	-0.23479128	1.07901717
O	74	1.8631022	-1.47518054	-2.31286798
C	75	-2.35570252	2.14086228	1.39059223
H	76	-1.77420782	2.98093338	0.99657024
C	77	3.61987719	-2.82918939	-1.2565624
H	78	4.47282248	-2.43128514	-0.69172549
N	79	-3.4857317	1.93302562	0.48549397
N	80	3.04953593	-3.94503106	-0.51000766
H	81	2.40407128	-4.59309835	-0.96130281
C	82	4.08479715	-3.25824969	-2.66621409

H	83	3.21299058	-3.52510546	-3.27668768
C	84	4.82627311	-2.11487505	-3.3707866
H	85	5.71501972	-1.81288696	-2.80596194
H	86	5.15172311	-2.42282802	-4.3704296
H	87	4.1896193	-1.2338188	-3.49362402
C	88	5.00457791	-4.48316007	-2.61397052
H	89	5.37613761	-4.73484164	-3.61322481
H	90	4.4706947	-5.3612469	-2.23891749
H	91	5.86955789	-4.30227979	-1.9671104
C	92	-2.85623379	2.49713055	2.80844222
H	93	-3.62399109	3.27579797	2.69724567
C	94	-1.74803749	3.09726904	3.67765541
H	95	-1.25609669	3.93084528	3.16563228
H	96	-2.1646865	3.48397529	4.6142222
H	97	-0.98720809	2.35596654	3.93969706
C	98	-3.51303163	1.32081132	3.53761138
H	99	-4.3308849	0.8919982	2.95040505
H	100	-3.93372794	1.65048658	4.49396236
H	101	-2.79176612	0.52588353	3.75241669
C	102	2.98975515	-3.97409091	0.86516206
H	103	-4.08179551	1.12444438	0.66794332
C	104	-3.36660563	2.15880826	-0.87243707
O	105	3.51288615	-3.11701086	1.57589927
O	106	-2.39076732	2.69640564	-1.39102995
C	107	-4.59929769	1.70086203	-1.67820346
H	108	-4.30164971	1.59970712	-2.72849399
C	109	2.21303549	-5.17811253	1.42906063
H	110	1.61821916	-4.80183395	2.27104837
N	111	-5.00827363	0.36029787	-1.17920545
N	112	1.24360872	-5.68307813	0.4154719
C	113	-5.75372555	2.701539	-1.51916628
C	114	3.17254566	-6.25610296	1.96074942
H	115	3.73709564	-5.83672699	2.8049522
H	116	-5.79535649	0.03531013	-1.74181501
H	117	-4.24552381	-0.28281012	-1.45286572
H	118	0.48217812	-4.98179171	0.37078905
H	119	0.77784855	-6.50227327	0.80569279
H	120	2.98849672	6.61946171	1.52965491
C	121	4.66088652	6.10131781	2.77656863
C	122	7.10245095	6.71764355	4.00843118
C	123	5.08811347	5.38677933	3.90412554
C	124	5.4718631	7.12730708	2.27321704
C	125	6.68693036	7.4335352	2.88732498
C	126	6.30354712	5.69510853	4.51682616
H	127	4.47535653	4.58713989	4.31465549
H	128	5.16157438	7.69262759	1.39664448
H	129	7.30905278	8.23184849	2.49101495
H	130	6.62682762	5.13824892	5.39252796

H	131	8.0481586	6.95790548	4.48704924
H	132	-1.87221719	-3.74826489	3.31145298
C	133	-3.86778916	-3.93278223	4.0170831
C	134	-5.79041922	-3.62740348	6.03475412
C	135	-4.71481999	-4.99576664	4.35768116
C	136	-3.99291211	-2.71668129	4.7029121
C	137	-4.95078088	-2.5651253	5.7060055
C	138	-5.67188301	-4.84222277	5.36203343
H	139	-4.63578616	-5.95113573	3.84415475
H	140	-3.34270471	-1.87931834	4.45876128
H	141	-5.04073749	-1.61799138	6.2314845
H	142	-6.32501119	-5.67149713	5.62118349
H	143	-6.53558822	-3.50920594	6.81697964
H	144	-6.6731565	2.28716709	-1.95477525
H	145	-5.98062613	2.8729954	-0.45844382
C	146	-5.47694673	4.02595869	-2.19392494
C	147	-4.92007553	6.46874783	-3.45229767
C	148	-5.03601497	5.12626853	-1.44655479
C	149	-5.63259388	4.16337802	-3.58006014
C	150	-5.35555533	5.38003677	-4.20517369
C	151	-4.76132124	6.34211954	-2.07384154
H	152	-4.89686765	5.04157292	-0.37088395
H	153	-5.97010185	3.32282136	-4.18222146
H	154	-5.47886677	5.47832692	-5.28046433
H	155	-4.41889754	7.18977597	-1.48627466
H	156	-4.70349349	7.41551094	-3.93968498
H	157	2.59121399	-7.08242509	2.39210772
C	158	4.15633018	-6.83202223	0.96783378
C	159	6.00018799	-7.9104817	-0.85244598
C	160	5.47724555	-6.36281151	0.92775645
C	161	3.77090863	-7.84370123	0.07880504
C	162	4.6884673	-8.3797553	-0.82652986
C	163	6.39429762	-6.901537	0.02377311
H	164	5.80073677	-5.57142608	1.60099813
H	165	2.75062585	-8.21936647	0.08385449
H	166	4.37788695	-9.16317128	-1.51253829
H	167	7.41536186	-6.53038021	0.00184649
H	168	6.7135137	-8.32789884	-1.5577239

- Lowest energy conformation for [**6f·6f**] dimer.

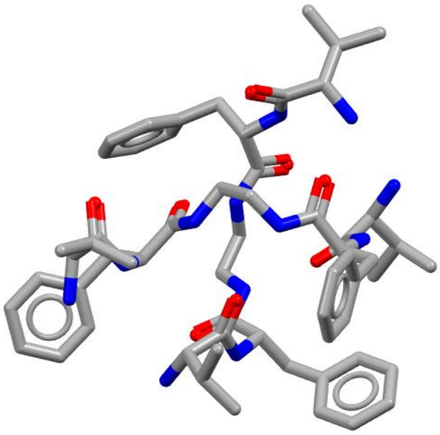

Cartesian coordinates (168 atoms), E (298 K) = 561.21 kJ/mol; Eaq (298 K) = 221.23 kJ/mol

C	1	-1.77004874	0.40842475	-2.24486284
H	2	-2.84426189	0.4326522	-2.45698112
H	3	-1.23174844	0.59633922	-3.17993103
C	4	-1.42990062	1.47221852	-1.19787881
H	5	-1.82764285	1.19060923	-0.21919222
H	6	-1.86753143	2.43472124	-1.48316857
N	7	0.00276472	1.67991286	-1.07746246
H	8	0.55840432	1.68165447	-1.92513557
N	9	-1.40483229	-0.92690847	-1.8045071
H	10	-0.40440027	-1.14411721	-1.78685019
C	11	0.55686854	2.36190226	-0.01441851
C	12	-2.26633876	-1.68121559	-1.03943811
O	13	-0.05377661	2.57924741	1.02998695
O	14	-3.42895493	-1.33479361	-0.81258044
C	15	2.0638029	2.64849014	-0.18059971
H	16	2.56584857	1.71052644	0.07797242
C	17	-1.70457648	-3.02119007	-0.5241505
H	18	-0.62719961	-2.93212872	-0.36466667
N	19	2.44434288	2.88615116	-1.57799918
N	20	-2.35260009	-3.2901167	0.76314176
H	21	-3.36326475	-3.43385719	0.76405908
C	22	-2.0374329	-4.14848028	-1.50922922
H	23	-3.11541933	-4.15206099	-1.72278492
C	24	2.52941933	3.74778659	0.79218049
C	25	-1.92231443	-2.70700843	1.93489821
H	26	3.37162037	2.57366721	-1.86001383
C	27	2.0898861	4.04681739	-2.23676818
O	28	-0.82420807	-2.169695	2.05861579
O	29	1.17158237	4.77812287	-1.88563354
C	30	2.9527436	4.31927145	-3.49018798
H	31	3.11677754	5.40419106	-3.51494084
C	32	-2.93601639	-2.84326049	3.09949521
H	33	-2.92876142	-1.89380145	3.6441853
N	34	4.28660742	3.67670707	-3.32473477

N	35	-4.30689452	-3.02422982	2.54574498
C	36	2.21229763	3.87919308	-4.77289018
H	37	1.19525067	4.29235005	-4.73158109
C	38	2.88223885	4.45079941	-6.02693023
H	39	3.88071115	4.03014647	-6.18348495
H	40	2.28470837	4.22775618	-6.91761092
H	41	2.97674638	5.5394624	-5.95632321
C	42	2.07326155	2.36005008	-4.91400131
H	43	3.04961792	1.8712673	-4.99117107
H	44	1.50634388	2.10929181	-5.81741361
H	45	1.53726832	1.9255895	-4.06559153
C	46	-2.58826525	-3.99785501	4.06618393
H	47	-3.36061036	-4.01503992	4.84811358
C	48	-2.58643322	-5.3812256	3.40774981
H	49	-2.39054955	-6.15935399	4.15375315
H	50	-1.81514653	-5.46300979	2.63667684
H	51	-3.55349834	-5.60737907	2.94793502
C	52	-1.2541409	-3.75536224	4.77935435
H	53	-1.23801005	-2.76769229	5.25190087
H	54	-1.09916349	-4.50189321	5.56597524
H	55	-0.40499775	-3.82069228	4.09245933
H	56	4.85131464	3.8943978	-4.14682451
H	57	4.76526268	4.147417	-2.54890479
H	58	-4.95390289	-3.11405254	3.33093065
H	59	-4.59288459	-2.13806898	2.09936766
C	60	1.28352906	0.52025725	3.59140951
H	61	1.98231369	0.20721732	4.37492888
H	62	1.67351363	1.44334626	3.14926663
C	63	-0.1032785	0.74665943	4.1940376
H	64	-0.03331578	1.44729798	5.03310683
H	65	-0.52127587	-0.19211103	4.57436236
N	66	-1.02712022	1.32419008	3.24089806
H	67	-0.65371158	1.96995127	2.53617615
N	68	1.2931704	-0.51739556	2.57836467
H	69	0.4389647	-1.0665937	2.43201591
C	70	-2.38878121	1.29328803	3.41815445
C	71	2.36964381	-0.73218585	1.75335582
O	72	-2.93755522	0.62310967	4.29406823
O	73	3.38139252	-0.0286335	1.77980935
C	74	-3.18475861	2.1696103	2.43409926
H	75	-2.59321512	2.34228654	1.53193659
C	76	2.24846403	-1.9691543	0.83990285
H	77	1.21844724	-2.04726238	0.48581067
N	78	-4.37348548	1.41901815	2.04813478
N	79	3.12438042	-1.75479451	-0.3078973
H	80	4.10573038	-1.56014715	-0.10963583
C	81	2.65655235	-3.19974019	1.66140818
H	82	3.72586885	-3.15248305	1.91030734

C	83	-3.55927947	3.50604783	3.09013392
H	84	-4.2780999	4.04687485	2.46003477
C	85	2.67504364	-1.1048477	-1.43840886
H	86	-4.58327313	0.55979217	2.55131546
C	87	-4.9617484	1.56405415	0.8134318
O	88	1.48782004	-0.95703016	-1.71706443
O	89	-4.63483962	2.42412249	0.0003095
C	90	-6.10387235	0.55585011	0.55263016
H	91	-6.32904279	0.58000558	-0.52069982
C	92	3.82611824	-0.58185316	-2.33656608
H	93	3.50042164	0.3801809	-2.74749236
N	94	-5.59380009	-0.80226626	0.88675028
N	95	5.01897074	-0.31042699	-1.48701099
C	96	-7.36413118	0.90615514	1.37937711
H	97	-7.11209892	0.91348099	2.44819321
C	98	-7.88574103	2.30417128	1.0205987
H	99	-8.79461079	2.537132	1.5860709
H	100	-8.12384179	2.37620788	-0.04612066
H	101	-7.15040666	3.07891726	1.25936141
C	102	-8.4947486	-0.10936443	1.17229612
H	103	-8.749345	-0.20746068	0.11153278
H	104	-9.39843941	0.19966064	1.70904807
H	105	-8.22017041	-1.09824391	1.55237017
C	106	4.18737628	-1.54479304	-3.48719184
H	107	5.0712544	-1.13937378	-3.99930092
C	108	4.54375806	-2.96011537	-3.02429875
H	109	4.88763555	-3.56350651	-3.87167475
H	110	5.34803521	-2.94921181	-2.28222215
H	111	3.67938266	-3.47123483	-2.58932675
C	112	3.06906519	-1.60055035	-4.53082875
H	113	3.34542222	-2.25627414	-5.36338231
H	114	2.13584702	-1.97360117	-4.10279428
H	115	2.87421059	-0.60648314	-4.94422258
H	116	-6.26818024	-1.48280245	0.53232848
H	117	-4.77229348	-0.95101875	0.26845592
H	118	4.77967851	0.41758698	-0.80823961
H	119	5.74868019	0.09559538	-2.0742433
H	120	2.33520471	3.44240116	1.82844281
H	121	1.93568838	4.65895755	0.64589523
C	122	4.00237768	4.07245118	0.66047701
C	123	6.72648752	4.65644526	0.35089958
C	124	4.97619833	3.13985482	1.0412513
C	125	4.41027443	5.30734999	0.13660965
C	126	5.76645209	5.59662044	-0.0185283
C	127	6.33173038	3.43020974	0.8830697
H	128	4.68587866	2.18388411	1.4741502
H	129	3.67009658	6.0518855	-0.15192604
H	130	6.07397779	6.56003119	-0.41719295

H	131	7.07961418	2.70226832	1.18789334
H	132	7.78336604	4.88472505	0.2394228
H	133	-1.84069414	-5.11975048	-1.03420891
C	134	-1.27236529	-4.10808189	-2.80870732
C	135	0.12996912	-4.1018239	-5.23709223
C	136	0.0658492	-4.51651352	-2.85810221
C	137	-1.89464061	-3.67850971	-3.98854295
C	138	-1.19504746	-3.67351642	-5.19624958
C	139	0.75973602	-4.52352112	-4.06807054
H	140	0.57716329	-4.84054728	-1.95550152
H	141	-2.9304854	-3.34596235	-3.97609028
H	142	-1.68679391	-3.33940325	-6.1058951
H	143	1.79530294	-4.85188048	-4.09399316
H	144	0.67325873	-4.10226306	-6.17791961
H	145	2.13985375	-3.19689007	2.63077784
C	146	2.37095413	-4.51606018	0.97733018
C	147	1.84425553	-6.98077646	-0.25343988
C	148	3.3154274	-5.10045629	0.12411761
C	149	1.15532506	-5.17642647	1.19667626
C	150	0.89460302	-6.40469432	0.58793906
C	151	3.05260124	-6.32701695	-0.48840669
H	152	4.26451276	-4.60422501	-0.06587337
H	153	0.40583471	-4.73887493	1.85110914
H	154	-0.05011327	-6.91039037	0.77011636
H	155	3.79304405	-6.77382275	-1.14657332
H	156	1.64121522	-7.93683005	-0.72864952
H	157	-4.0834049	3.32718208	4.03865656
C	158	-2.37128495	4.40906306	3.32862667
C	159	-0.12002912	6.02727696	3.7475577
C	160	-1.83536179	4.56098605	4.61426939
C	161	-1.76829899	5.08080314	2.25683515
C	162	-0.64679501	5.88412178	2.46574264
C	163	-0.71495399	5.36741506	4.8215663
H	164	-2.28207244	4.04457554	5.46124961
H	165	-2.15790936	4.96662895	1.24638604
H	166	-0.18206588	6.38943386	1.62303362
H	167	-0.30466578	5.4784114	5.82159382
H	168	0.754536	6.65150135	3.90826041

- Lowest energy conformation for [**6b**-CO_2] symmetric dimer.

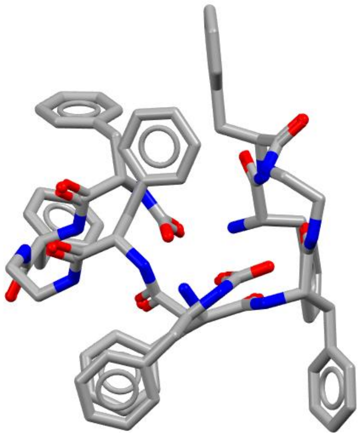

Cartesian coordinates (190 atoms), E (298 K) = -1425.94 kJ/mol; Eaq (298 K) = -1965.74 kJ/mol

1	C	-5.85932615	-0.87615703	-0.5986871
2	H	-6.62412105	-1.57422255	-0.9545943
3	H	-6.36229324	-0.02839371	-0.121427
4	C	-5.00135098	-0.42433356	-1.78172486
5	H	-5.65196828	-0.12820136	-2.61183106
6	H	-4.35383791	-1.23558569	-2.12518271
7	N	-4.15122315	0.69882944	-1.44668485
8	H	-3.27373957	0.49690845	-0.98092605
9	N	-5.09886255	-1.59511563	0.4079119
10	H	-4.74980546	-2.51887229	0.099459
11	C	-4.67253981	1.97609454	-1.36698306
12	C	-4.45790228	-1.00286287	1.47542369
13	O	-5.80568513	2.25907656	-1.74845484
14	O	-4.34010266	0.2157834	1.62021568
15	C	-3.70878258	3.02457682	-0.79333673
16	H	-4.27669569	3.94336339	-0.60084103
17	C	-3.85281109	-2.03060031	2.45284059
18	H	-4.30198851	-3.00601319	2.22237993
19	N	-3.23799348	2.53422001	0.51055705
20	N	-2.43278217	-2.21175206	2.16810407
21	H	-2.26488843	-2.71210722	1.2887696
22	C	-4.2224575	-1.67918876	3.9019574
23	H	-5.3119047	-1.5562391	3.9779365
24	C	-2.54479836	3.27953697	-1.76455441
25	C	-1.38442887	-1.44174756	2.6166071
26	H	-3.66642609	1.67440862	0.874277
27	C	-2.58110543	3.36732383	1.37801231
28	O	-1.5116828	-0.4811089	3.37069819
29	O	-2.28679258	4.53636833	1.11742918
30	C	-2.20681144	2.77593261	2.740864
31	H	-2.06971906	1.69867208	2.66441096
32	C	-0.01516767	-1.95028725	2.11212739
33	H	-0.0091347	-3.03955337	2.24750032

34	C	-3.29942925	3.14492632	3.75694172
35	H	-4.16630271	2.48614618	3.61356991
36	C	1.14006396	-1.31151754	2.88927892
37	C	4.58373925	-3.10817606	-2.56789338
38	H	4.86570444	-4.06109345	-3.02852301
39	H	4.82814012	-3.15739531	-1.50221441
40	C	5.34465642	-1.95652632	-3.23956413
41	H	5.1338721	-1.91971959	-4.31289012
42	H	6.42183032	-2.10863426	-3.11117897
43	N	4.99157071	-0.65456769	-2.70011162
44	H	4.24979095	-0.12569056	-3.16067833
45	N	3.14355242	-2.9596148	-2.66233623
46	H	2.60250845	-3.02505993	-1.80117515
47	C	5.4970596	-0.16344456	-1.52147955
48	C	2.45903156	-3.13617663	-3.84170607
49	O	6.36520459	-0.76730452	-0.89141145
50	O	3.02060189	-3.44239455	-4.89261985
51	C	4.99739091	1.25257946	-1.12234996
52	H	5.35173652	1.93982722	-1.90232479
53	C	0.9361494	-2.87517062	-3.77371629
54	H	0.59222863	-3.00502628	-4.80955391
55	N	3.53137315	1.3658297	-1.0688841
56	N	0.19965329	-3.89664055	-3.02194083
57	H	-0.70733212	-4.15895423	-3.39719168
58	C	0.64524946	-1.43430317	-3.31721017
59	H	1.5526618	-0.82524627	-3.38690848
60	C	5.62758191	1.64470161	0.2318531
61	C	0.31990592	-4.10600413	-1.66477874
62	H	3.14827827	1.87066709	-0.27552041
63	C	2.77453082	1.56784022	-2.20086126
64	O	1.15788422	-3.53264662	-0.96404509
65	O	3.17826113	1.25044291	-3.31987711
66	C	1.37176322	2.17966382	-1.95361244
67	H	0.64533813	1.3871836	-2.16898745
68	C	-0.65256289	-5.17786254	-1.10591996
69	H	-0.54437774	-5.20856145	-0.01341291
70	C	1.11158545	3.29474873	-2.99330669
71	C	-0.30641421	-6.56149843	-1.68764728
72	H	-0.37857914	-6.55689964	-2.78308895
73	H	-3.81780553	-0.70380536	4.18764159
74	C	-3.80421913	-2.73259133	4.90469081
75	C	-3.0193783	-4.69743582	6.74809418
76	C	-2.73779767	-2.50044648	5.78394953
77	C	-4.47479884	-3.96231446	4.96439458
78	C	-4.08285293	-4.93927426	5.88102337
79	C	-2.34727619	-3.47808691	6.70034303

80	H	-2.2019812	-1.5550942	5.7600828
81	H	-5.30950837	-4.16918251	4.29773885
82	H	-4.61006251	-5.88920132	5.91927025
83	H	-1.52079306	-3.28761651	7.37971933
84	H	-2.71783425	-5.45840884	7.46305971
85	H	1.28840281	-0.26569429	2.59497193
86	H	0.90270391	-1.27654386	3.96124239
87	C	2.42590042	-2.08674195	2.72752328
88	C	4.77141427	-3.57911732	2.39002694
89	C	3.38661601	-1.6882395	1.79109667
90	C	2.65293304	-3.24085561	3.49319375
91	C	3.82074035	-3.98379221	3.32266094
92	C	4.55534009	-2.43215122	1.62904607
93	H	3.23527847	-0.79964213	1.18097716
94	H	1.92354366	-3.57261852	4.22909487
95	H	3.99121249	-4.87792892	3.91653258
96	H	5.30542533	-2.12051105	0.90698107
97	H	5.68386188	-4.15405931	2.25402606
98	H	-1.68729892	3.71362847	-1.2365548
99	H	-2.16359481	2.34185629	-2.18958284
100	C	-2.92003168	4.22023742	-2.88462821
101	C	-3.62242317	5.97470955	-4.95304021
102	C	-2.780783	5.60499314	-2.71936303
103	C	-3.42597982	3.72573275	-4.09230491
104	C	-3.77392388	4.59975222	-5.12253254
105	C	-3.12725837	6.47751459	-3.75134693
106	H	-2.39988838	6.01027048	-1.78416674
107	H	-3.55654636	2.65587601	-4.23617969
108	H	-4.1668848	4.206661	-6.0561736
109	H	-3.01223827	7.5496294	-3.61754976
110	H	-3.89411204	6.65414263	-5.75622959
111	H	-3.6767812	4.15553107	3.54868148
112	C	-2.87013398	3.06983092	5.20439933
113	C	-2.01215169	2.95088093	7.87188435
114	C	-2.78558431	4.23634365	5.97796844
115	C	-2.52843798	1.84410192	5.78832664
116	C	-2.10051276	1.78524712	7.11487709
117	C	-2.35681529	4.17557379	7.30468262
118	H	-3.06058757	5.20357587	5.56089062
119	H	-2.59551101	0.92092393	5.21905764
120	H	-1.84535322	0.82704869	7.56167235
121	H	-2.30131753	5.08327838	7.90130611
122	H	-1.68694741	2.90310752	8.90841476
123	H	-1.05052508	-7.29811064	-1.35545052
124	C	1.06282365	-7.0446928	-1.26877852
125	C	3.6187695	-7.8637017	-0.45846447

126	C	1.25827622	-7.61504441	-0.00360068
127	C	2.16342872	-6.89114357	-2.12342585
128	C	3.4350509	-7.29800829	-1.71840032
129	C	2.53099198	-8.02354133	0.39796235
130	H	0.42073535	-7.74362588	0.6783636
131	H	2.04330774	-6.44494997	-3.10912339
132	H	4.28218017	-7.170213	-2.38757217
133	H	2.67490532	-8.4657193	1.38015185
134	H	4.60996556	-8.18000289	-0.14489062
135	H	0.33888776	-1.38739203	-2.26652916
136	C	-0.425181	-0.77742511	-4.15846043
137	C	-2.38567808	0.38288016	-5.78853595
138	C	-0.06713756	0.12642539	-5.16913476
139	C	-1.77674069	-1.08687335	-3.96747218
140	C	-2.75109835	-0.51167731	-4.78445029
141	C	-1.04462098	0.70769186	-5.97639736
142	H	0.97818489	0.38112189	-5.33766946
143	H	-2.07984912	-1.7804031	-3.18557641
144	H	-3.79845935	-0.76219693	-4.63875541
145	H	-0.75817199	1.40891234	-6.75593327
146	H	-3.14712029	0.82975618	-6.42219536
147	H	6.72102429	1.55564148	0.18434041
148	H	5.30604907	0.941209	1.01129262
149	C	5.28924405	3.05653614	0.66005158
150	C	4.60108839	5.67289019	1.39496377
151	C	5.88947077	4.1528776	0.02750668
152	C	4.34520045	3.28695673	1.67095755
153	C	4.00126073	4.58965223	2.03303777
154	C	5.54694058	5.45439832	0.39549126
155	H	6.62307794	4.0017699	-0.76114762
156	H	3.85894486	2.45379938	2.17487048
157	H	3.26147803	4.75732664	2.81014768
158	H	6.01174118	6.2989949	-0.10600817
159	H	4.33065811	6.68779393	1.67248321
160	H	0.04912965	3.56021363	-2.97378959
161	H	1.29203041	2.91850337	-4.0087834
162	C	1.91939223	4.56082624	-2.79872926
163	C	3.4091885	6.9078758	-2.42485502
164	C	3.20936748	4.67717962	-3.33285042
165	C	1.38390374	5.63857375	-2.07890124
166	C	2.12624008	6.8049461	-1.89155819
167	C	3.95018446	5.84496304	-3.14553641
168	H	3.64390783	3.86165916	-3.90774918
169	H	0.38068596	5.57381727	-1.66273634
170	H	1.7006068	7.63466639	-1.33427338
171	H	4.94856764	5.92522652	-3.56676289

172	H	3.98604282	7.81733406	-2.28275886
173	N	1.1445674	2.64012845	-0.57750514
174	C	0.73190812	1.84359647	0.37015616
175	O	0.63959453	2.40109183	1.49680333
176	O	0.4696475	0.64383587	0.1020135
177	N	-2.04767574	-4.81596247	-1.39771322
178	C	-2.56966332	-3.7553371	-0.82900103
179	O	-1.78584004	-2.92482504	-0.29050001
180	O	-3.82096413	-3.70846811	-0.82017494
181	N	0.14690921	-1.72486493	0.66366755
182	H	0.92700721	-2.24165358	0.23680955
183	H	0.2476039	-0.71428411	0.39488838
184	N	-0.8847358	3.33839109	3.14726189
185	H	-0.84096949	4.34646889	2.97946383
186	H	-0.57751293	3.07846728	4.0840035
187	H	-2.69491402	-5.54799326	-1.65483567
188	H	1.45465701	3.57377751	-0.34916837
189	H	-0.16349184	2.94102988	2.48326351
190	H	-0.68727405	-2.07734435	0.13727042
